# Supplementary material for: Multi-functional soft-bodied jellyfish-like swimming
Source: Nat Commun. 2019 Jul 2;10:2703. doi: 10.1038/s41467-019-10549-7 (PMC6606650; doi:10.1038/s41467-019-10549-7)
Supplement: Supplementary file 1 — Supplementary Information [file 41467_2019_10549_MOESM1_ESM.pdf]

Supplementary Information

Multi-functional soft-bodied jellyfish-like swimming

Ziyu Ren*, Wenqi Hu*, Xiaoguang Dong, Metin Sitti#

[3]Physical Intelligence Department, Max Planck Institute for Intelligent Systems, 70569 Stuttgart, Germany

*Equally contributing authors
#Correspondence to: sitti@is.mpg.de

Figures

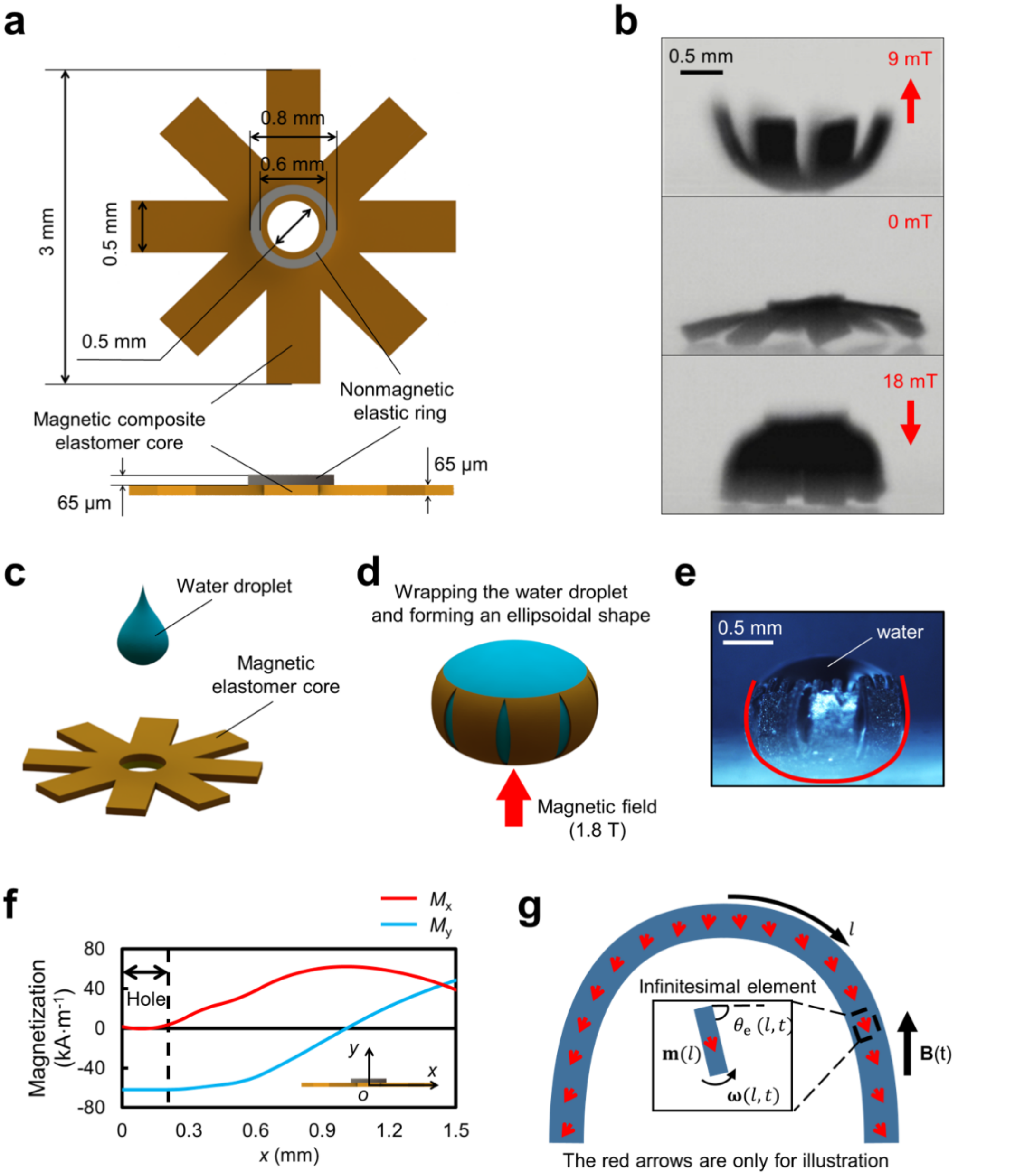

Supplementary Figure 1. Design, deformation, and fabrication of the magnetic composite elastomer core. a, Design parameters. **b**, The deformation of the core under the external **B** field. **c**, A water droplet is dropped on the magnetic composite elastomer core using a pipette. **d**, The core automatically wraps the water droplet to form an ellipsoidal shape. After the water is frozen, the core is magnetized under a uniform **B** field of 1.8 T. **e**, The side view of a core wrapping the water droplet. **f**, The magnetization profile of the core. The double-headed arrow indicates the region of the hole. **g**, Illustration for calculating the work done by magnetic torque. Note the red arrows are for illustration purpose and do not indicate the actual magnetization profile.

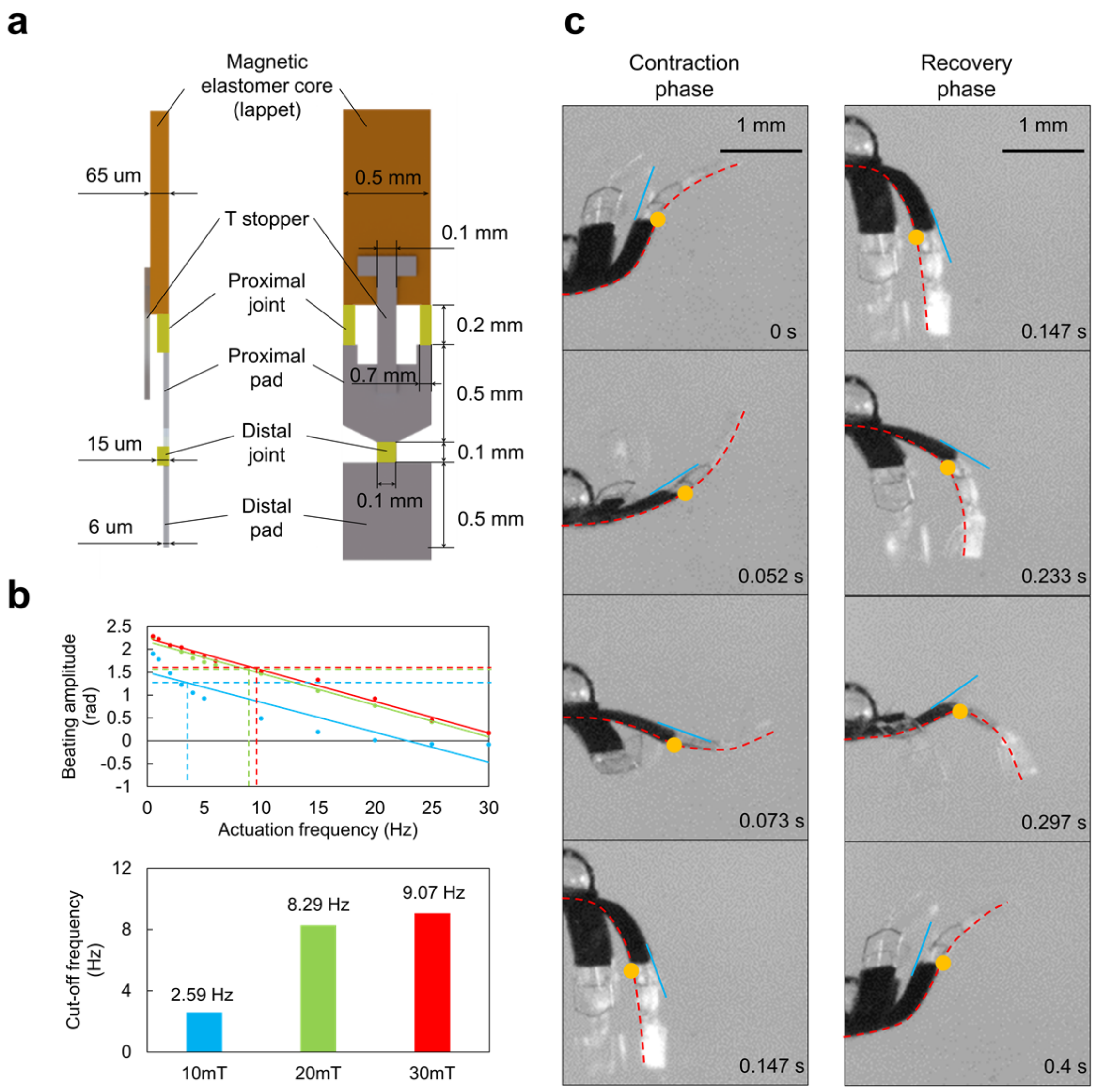

Supplementary Figure 2. Design and performance of the lappet. a, Detailed design of the passive lappet. Sketch of the geometry is not to scale for representation purposes. **b**, The cut-off frequency of the lappet. In the upper panel, beating amplitudes are measured under sinusoidal magnetic fields with different actuation amplitudes and frequencies. Five measurements are conducted at each data point. Linear least square regression is used to find the cut-off frequency. The horizontal dashed lines indicate the beating amplitude that is 0.707 times that achieved under 0.5 Hz. The lower panel shows the cut-off frequency under the sinusoidal magnetic fields with different amplitudes. See "Methods: Passive lappet" for the definition of the cut-off frequency. **c**, The proximal joint bends less in contraction phase than in the recovery phase. The positions of the proximal joint are marked with yellow dots. The body profiles are outlined by red dashed lines. The positions of the T stopper are marked by solid blue lines.

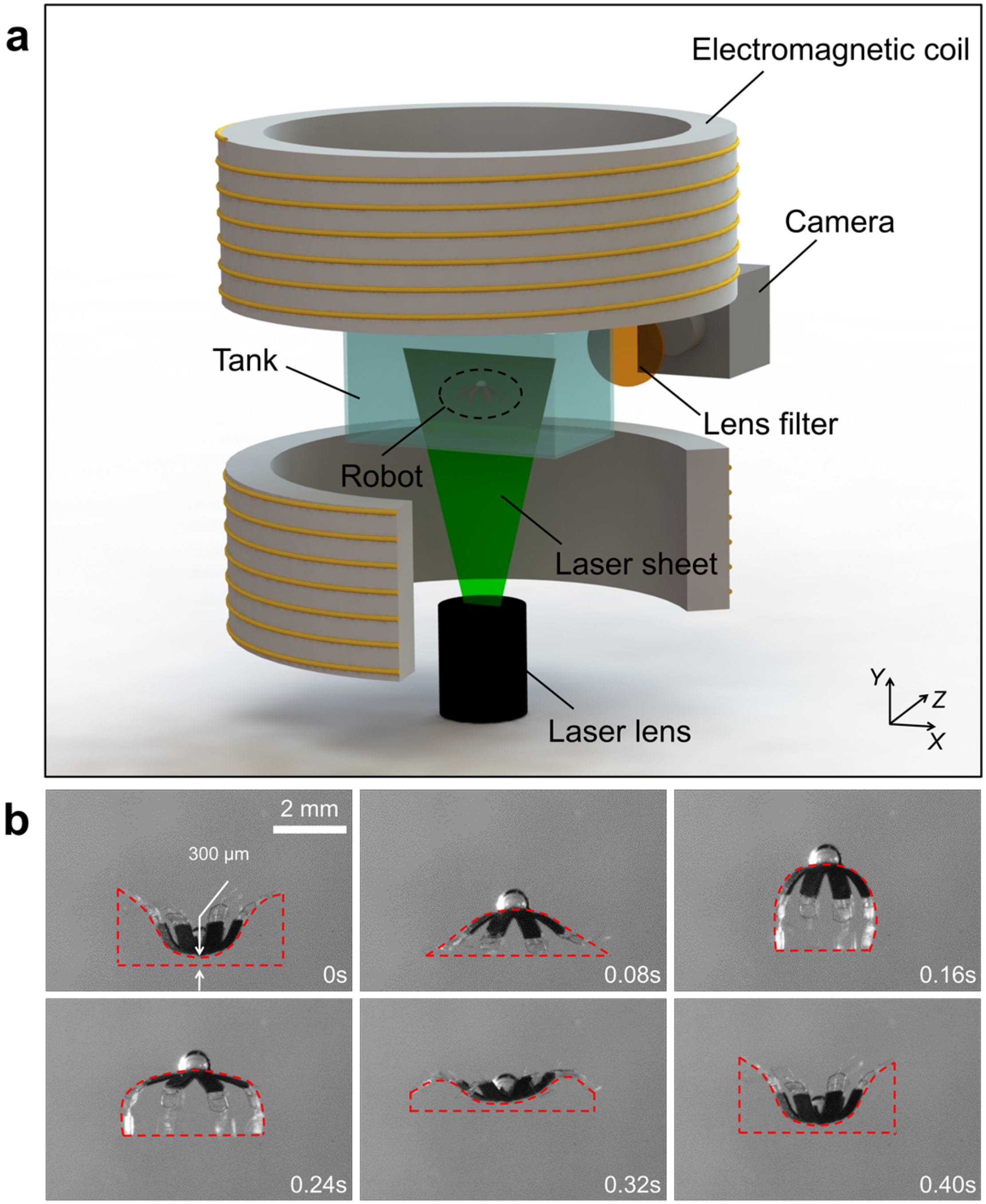

Supplementary Figure 3. Experimental setup and the definition of the sub-umbrella region. a, The Helmholtz coil system and the PIV setup. Sketch of the geometry is not to scale and only for representation purpose. **b**, The definition and variation of the sub-umbrella region (marked with red dashed curves) in one cycle. The motion sequence is from Mode A.

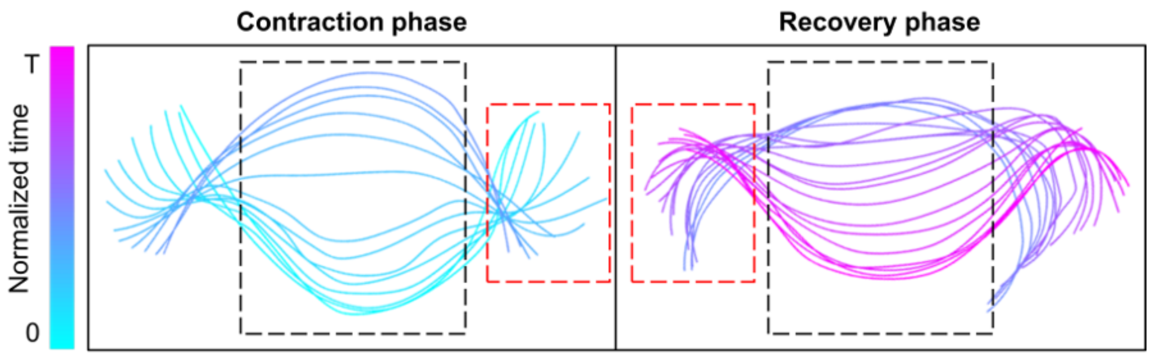

Supplementary Figure 4. The body profiles extracted from the real jellyfish ephyra. The black dashed boxes mark out the deformation of the central part of the body. The red dashed boxes mark out the deformation of the lappet. The video used for extracting body profiles is from Feitl *et al.*[1]; permission is conveyed through Copyright Clearance Center, Inc.

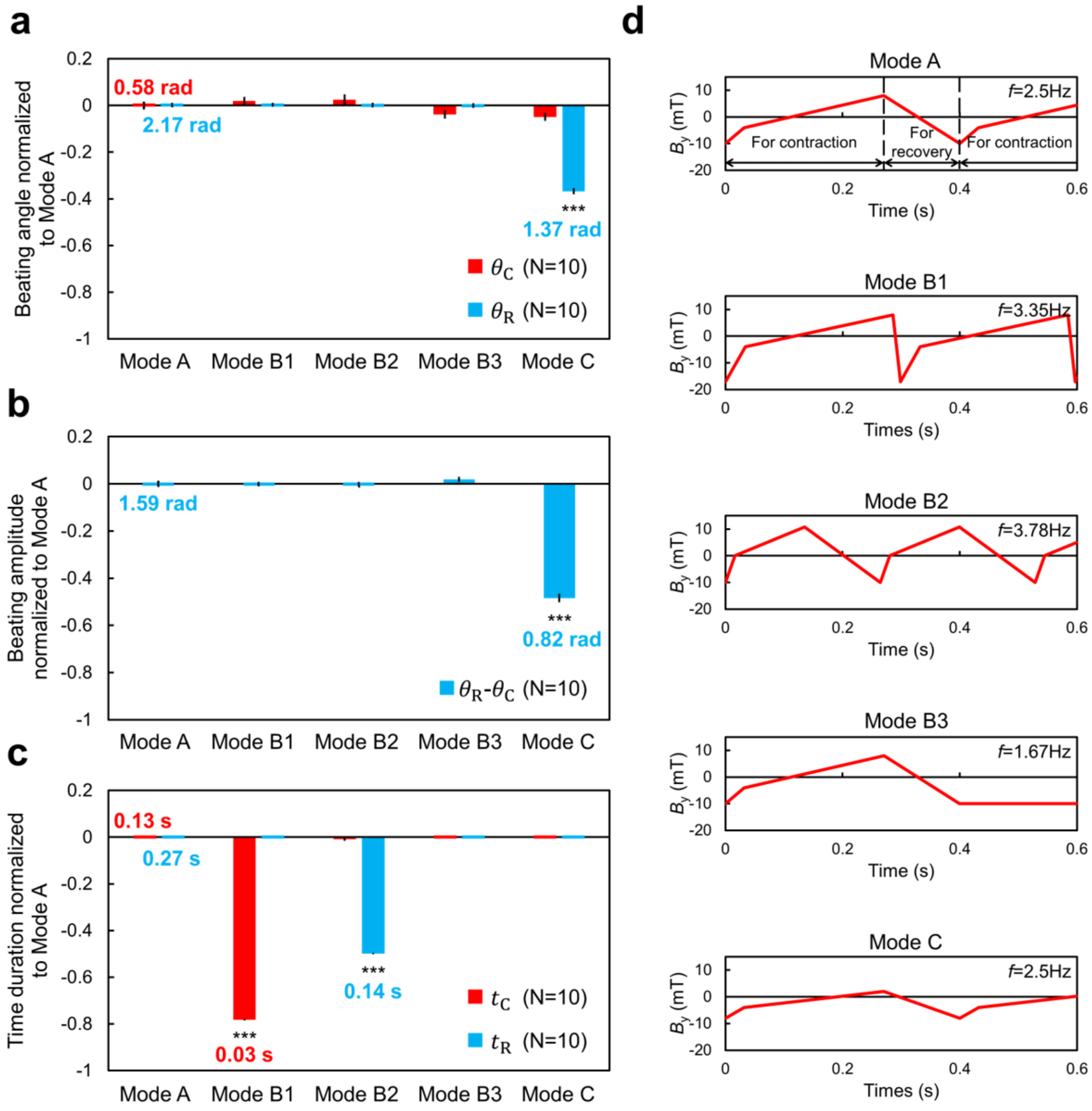

Supplementary Figure 5. The achieved kinematic parameters and the control signals applied. a-c, The comparison of the kinematic parameters among different basic swimming modes. All of the parameters are experimentally measured. The error bars represent the standard error of the mean. N is the number of trials. **d**, The control signals used to achieve five basic swimming modes.

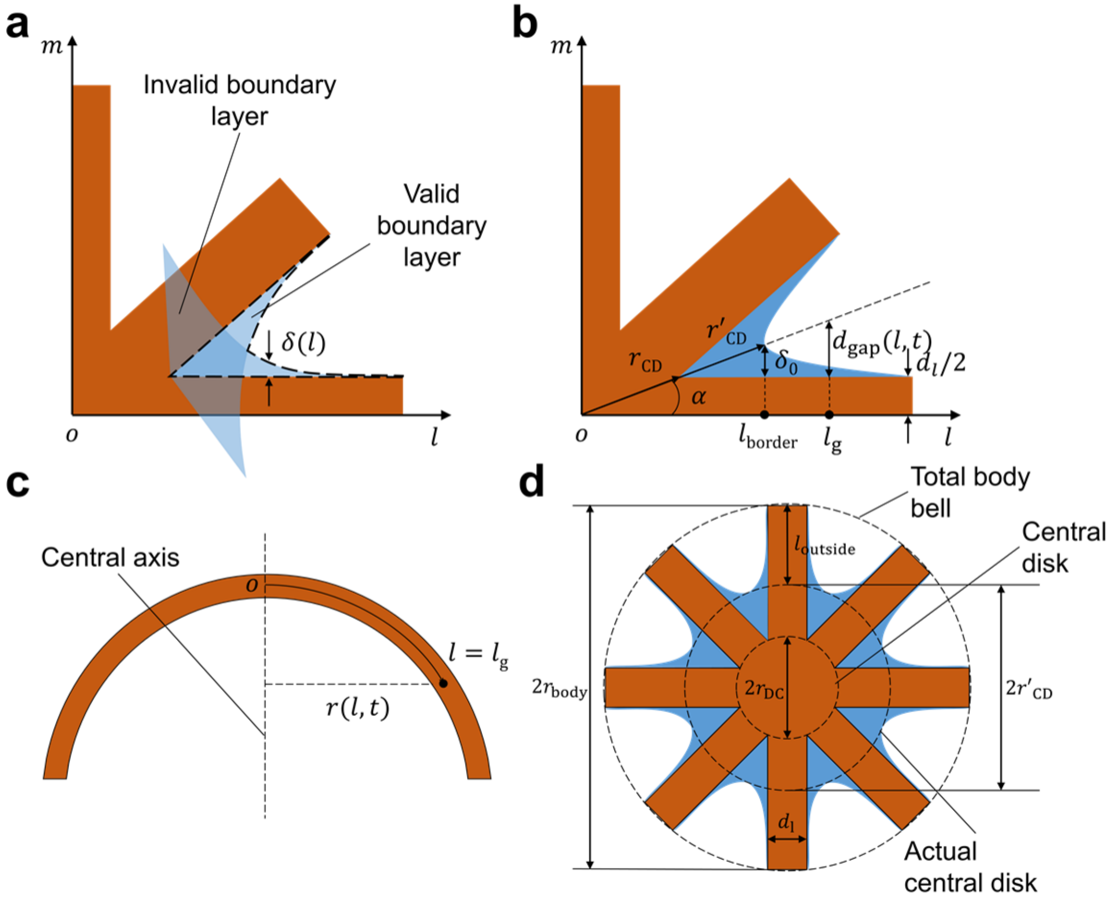

Supplementary Figure 6. Illustration of the calculation on effective boundary coverage (BC) rate. a, The calculation with Supplementary Equation 7 can generate unrealistic boundary layer that covers the core. Therefore, only the boundary layer (marked with black dashed curves) filling the gap should be considered (valid boundary layer). **b**, The boundary layer increases the radius of the effective central disk from r_{CD} to r'_{CD}. **c**, $r(l,t)$ is the distance between a given point on the lappet and the central axis of the body. It is directly measured from the video. **d**, The effctive BC rate is enlarged by the boundary layer within the gaps.

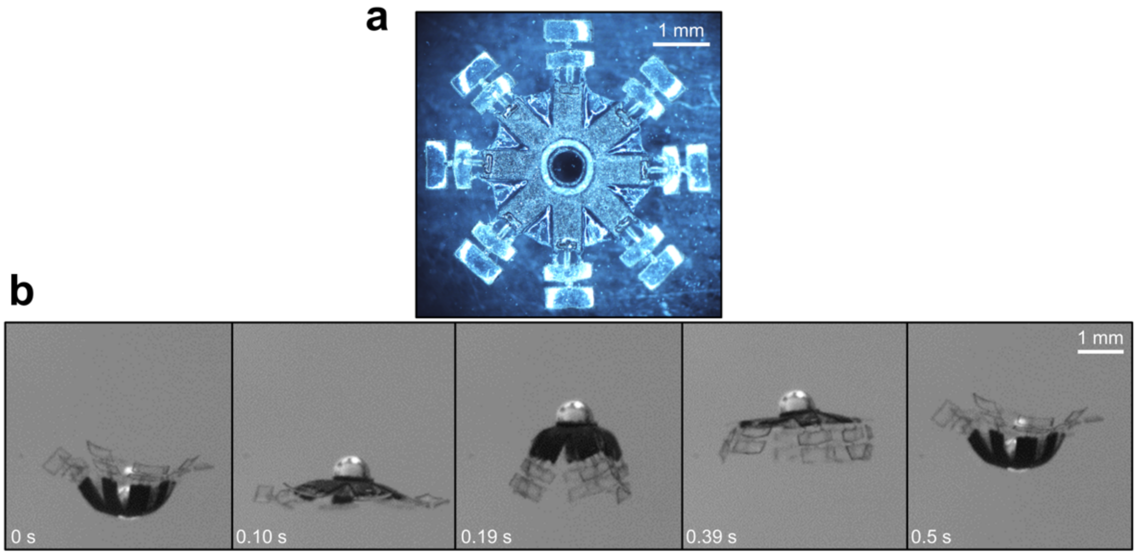

Supplementary Figure 7. The robotic jellyfish with a larger BC rate. a, The BC rate of the newly-designed robot can reach 60.87% without considering the boundary layer. **b**, The motion sequence of one beating cycle.

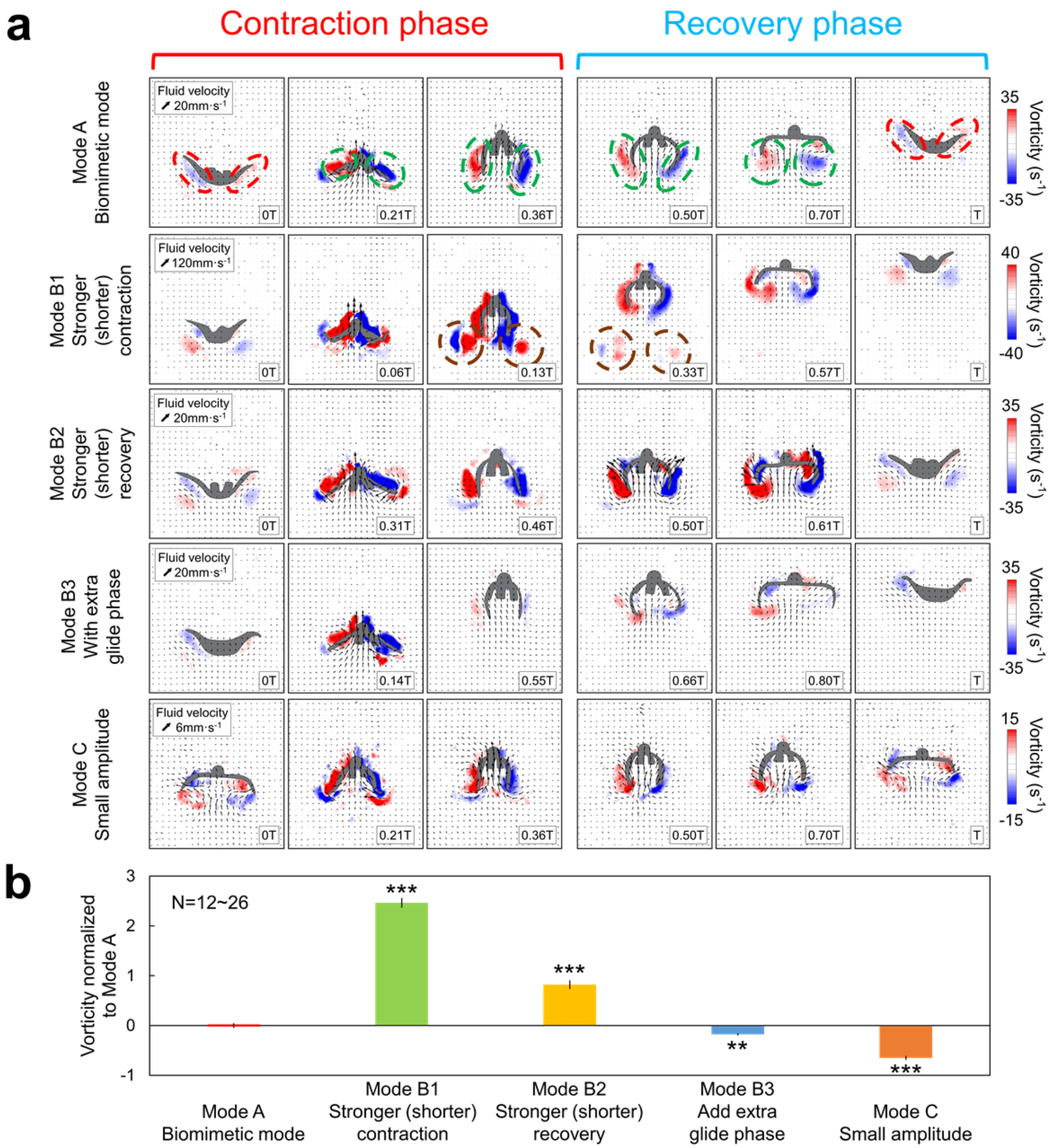

Supplementary Figure 8. General PIV results to compare the velocity and vorticity fields among different swimming modes. a, The sequence of the velocity and vorticity fields of different swimming modes in one cycle. The time stamps are normalized to one period (represented by T). The starting vortexes are marked with red dashed circles. The stopping vortexes are marked with green dashed circles. The vortexes shedding from the robot body in Mode B1 are marked with brown dashed circles. **b**, The circulation of the stopping vortex measured when the magnetic core recovers to be flat. The error bars represent the standard error of the mean. N is the number of the measured vortexes.

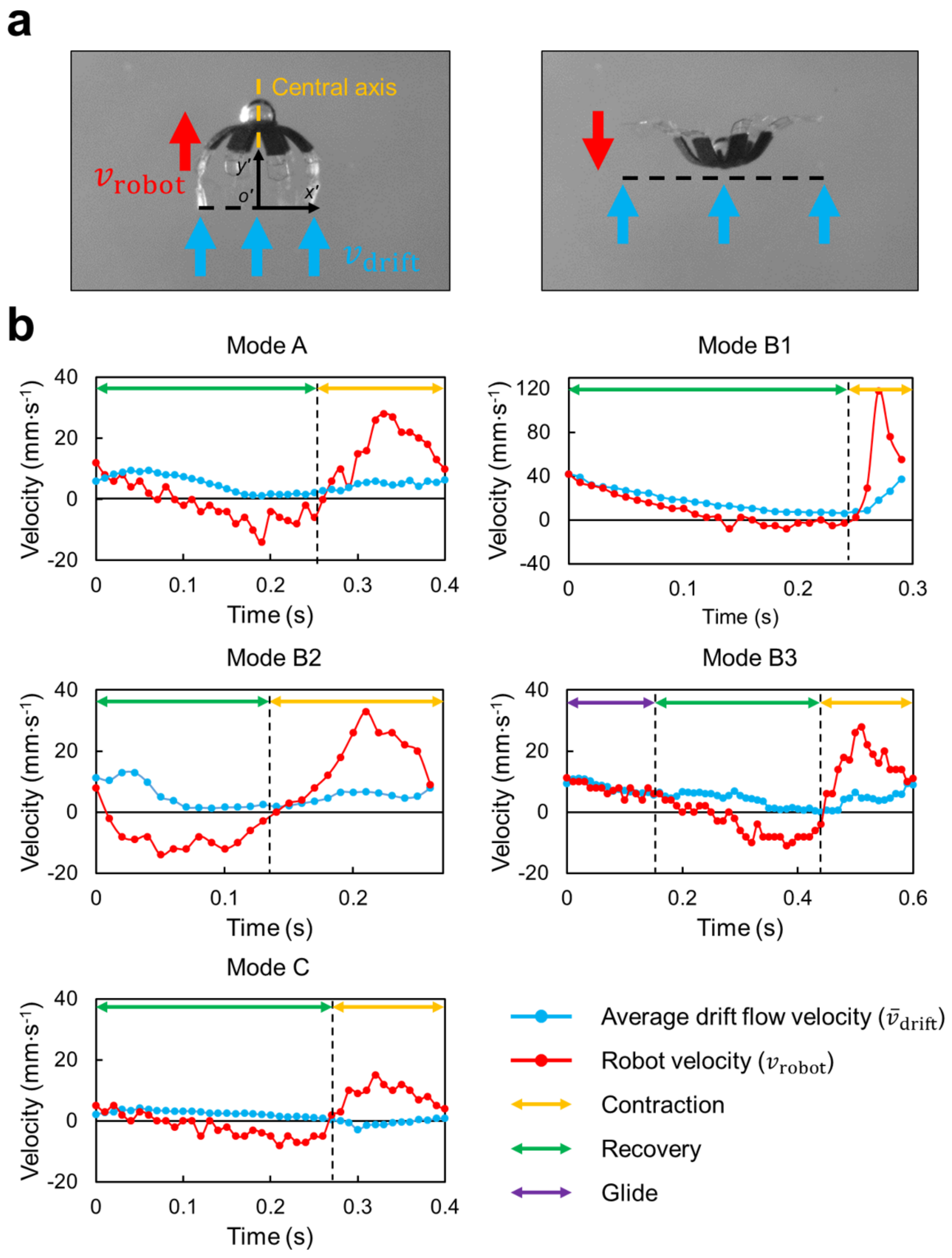

Supplementary Figure 9. Illustration of the calculation of Q_{exchange}. a, The coordinate frames used for calculation when the lappet tip is lower than the center bottom of the robot (left panel) and when the lappet tip is higher than the center bottom of the robot (right panel). The reference line (black dashed line) is the bottom boundary of the sub-umbrella region. The *x'*-axis and *y'*-axis of the body frame are along the reference line and the central axis, respectively. **b**, v_{robot} and $\bar{v}_{\text{drift}}$ (v_{drift} averaged along the reference line) of the five basic swimming modes.

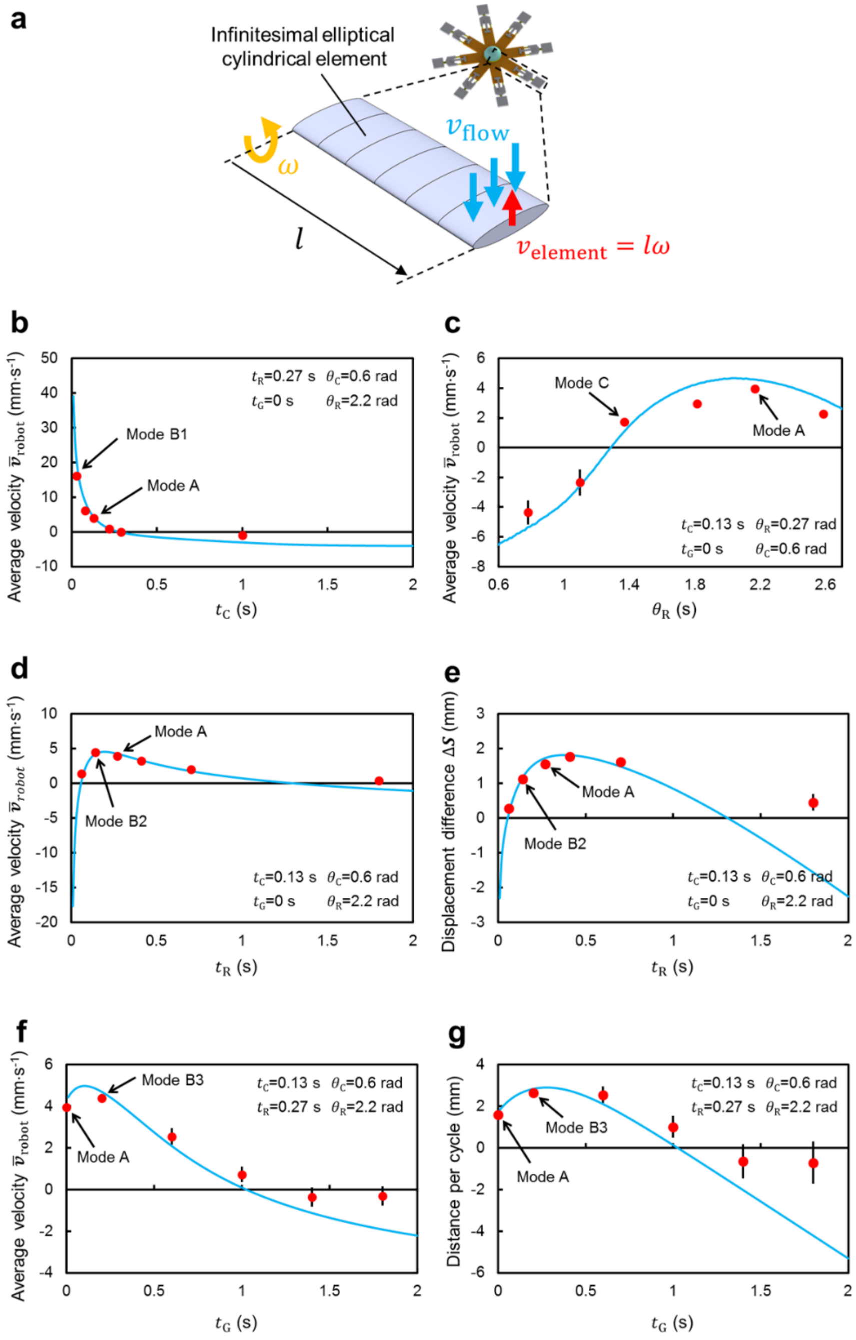

Supplementary Figure 10. The impact of changing the kinematic parameters on propulsion performance investigated through a dynamic model. a, Each lappet of the robot is modeled as a linear array of elliptical cylinders. **b-g**, The variation of the propulsion performance when changing the key kinematic parameters. The blue curves are the predictions from the simple dynamic model, and the red points indicate the experiment results. $\Delta S=S_C-S_R$ in **e** is the difference between the displacements during the contraction and recovery phases. See Supplementary Note 10 for more details.

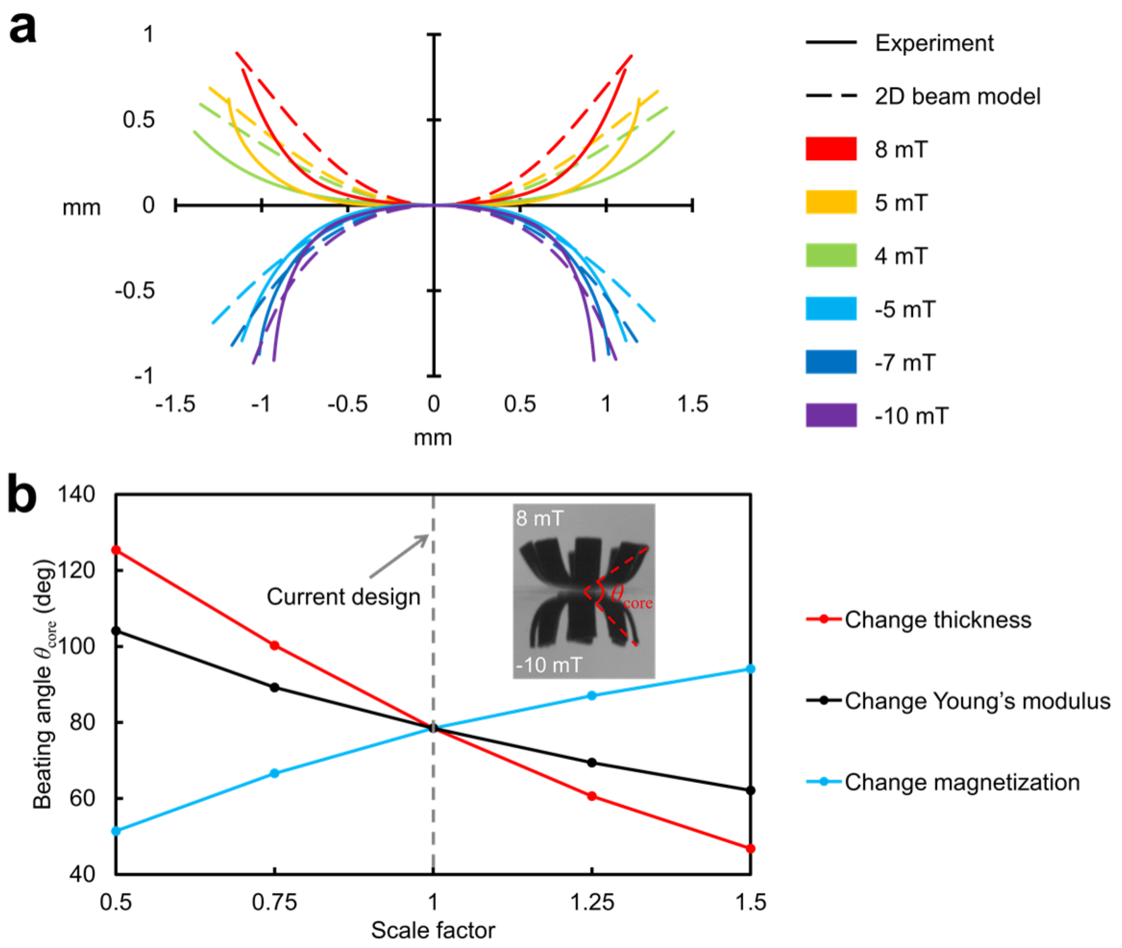

Supplementary Figure 11. The analysis of the core deformation at static condition. a, The comparison of the core deformation between the experiment results and the predictions of the 2D beam model. **b**, The relation between the design parameters and the beating angle, θ_{core}. θ_{core} is defined in the inset. Note 8 mT and -10 mT are, respectively, the peak and valley values of the control signal of Mode A. The dependent parameters are scaled up and down relative to the current design value.

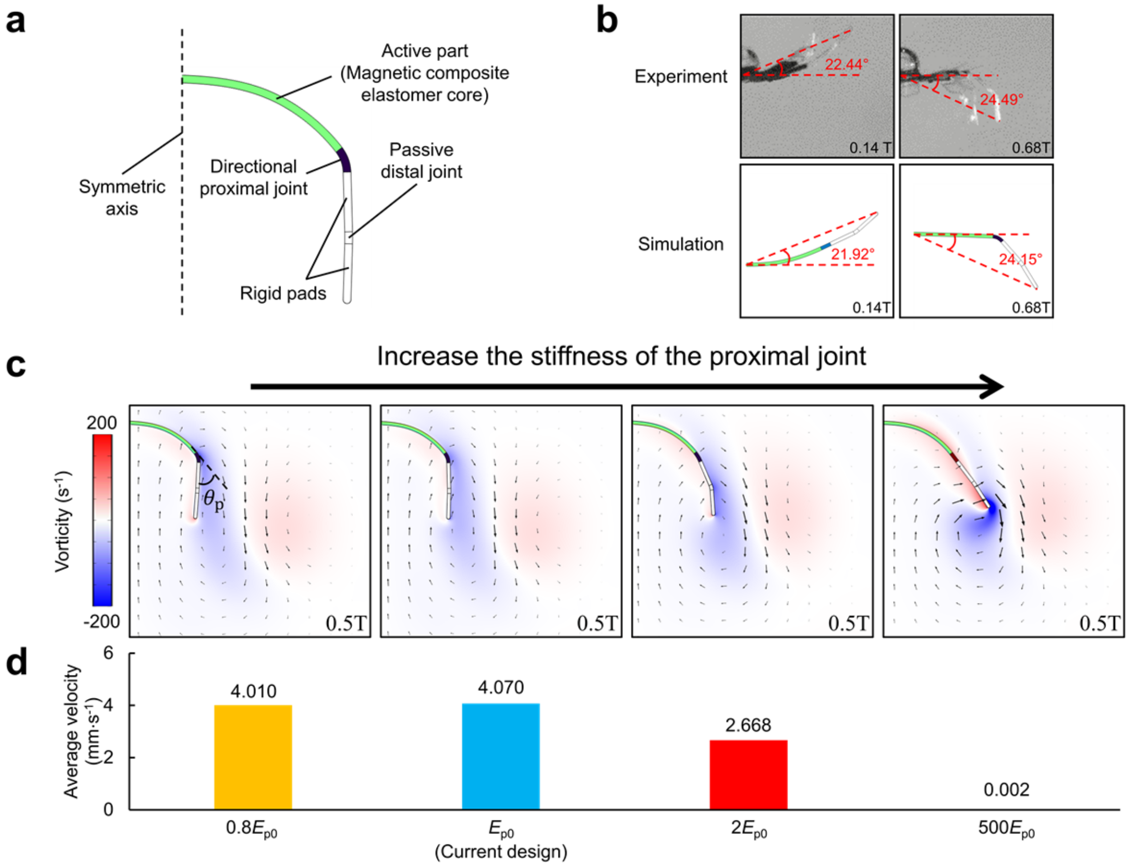

Supplementary Figure 12. The influence of changing the stiffness of the proximal joint. **a**, Half of the virtual robot used in the 2D simulation. **b**, The comparison between the kinematics achieved in experiment and in simulation after tuning E_{p} and E_{d} to be E_{p0} and E_{d0}, in order to make the achieved kinematics in the simulation match that of Mode A. **c**, The influence of the proximal joint stiffness on deformation amplitude (θ_{p}). **d**, The influence of the proximal joint stiffness on average swimming velocity.

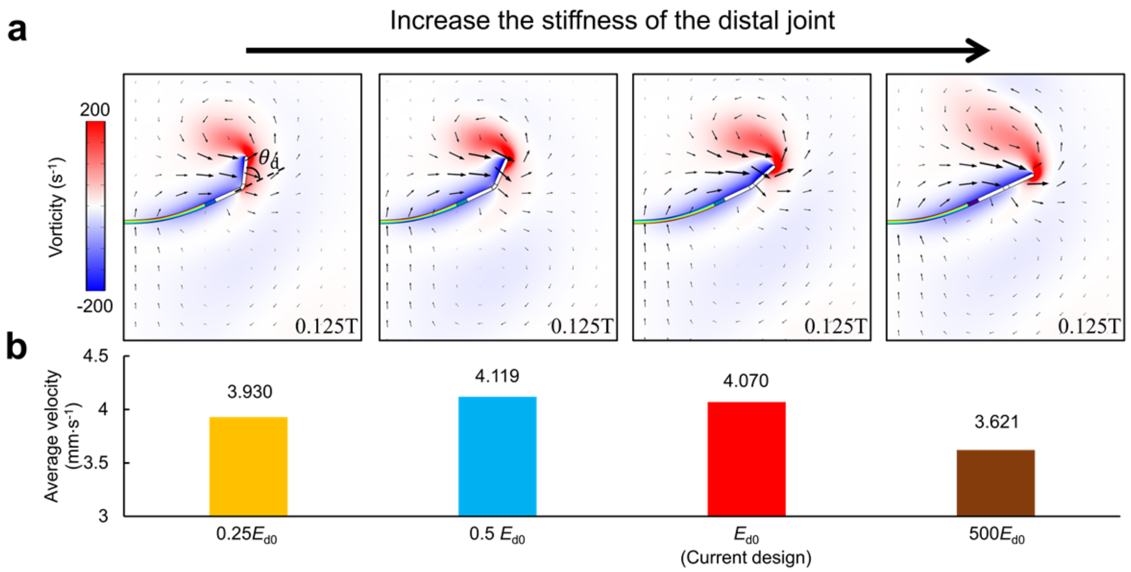

Supplementary Figure 13. The influence of changing the stiffness of the distal joint. **a**, The influence of the distal joint stiffness on deformation amplitude (θ_d). **b**, The influence of the distal joint stiffness on average swimming velocity.

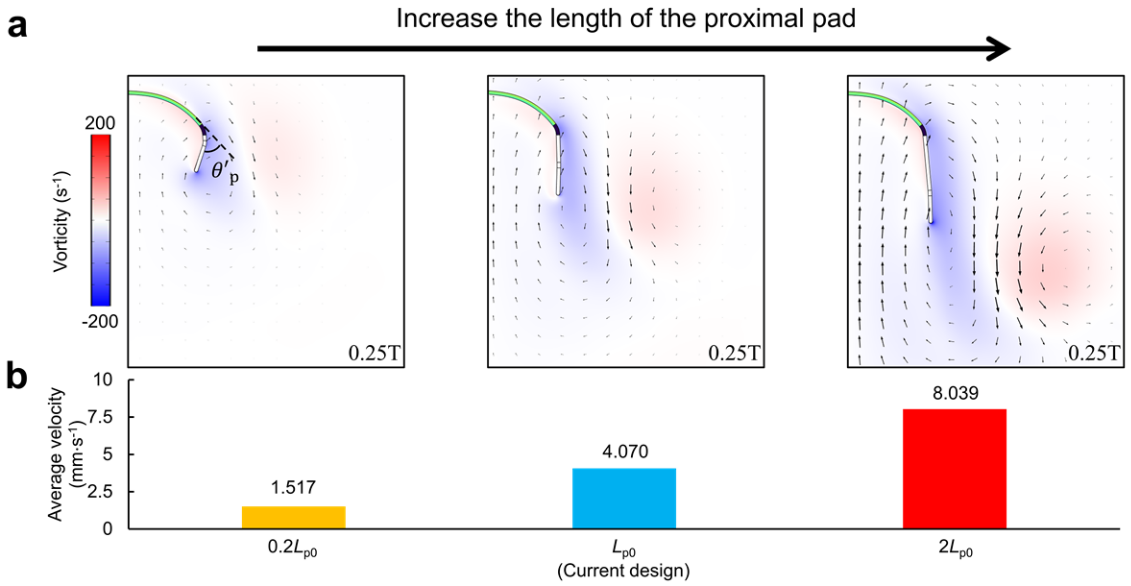

Supplementary Figure 14. The influence of changing the length of the proximal pad. **a**, The influence of the proximal pad length on deformation amplitude (θ'_{p}). **b**, The influence of the proximal pad length on average swimming velocity.

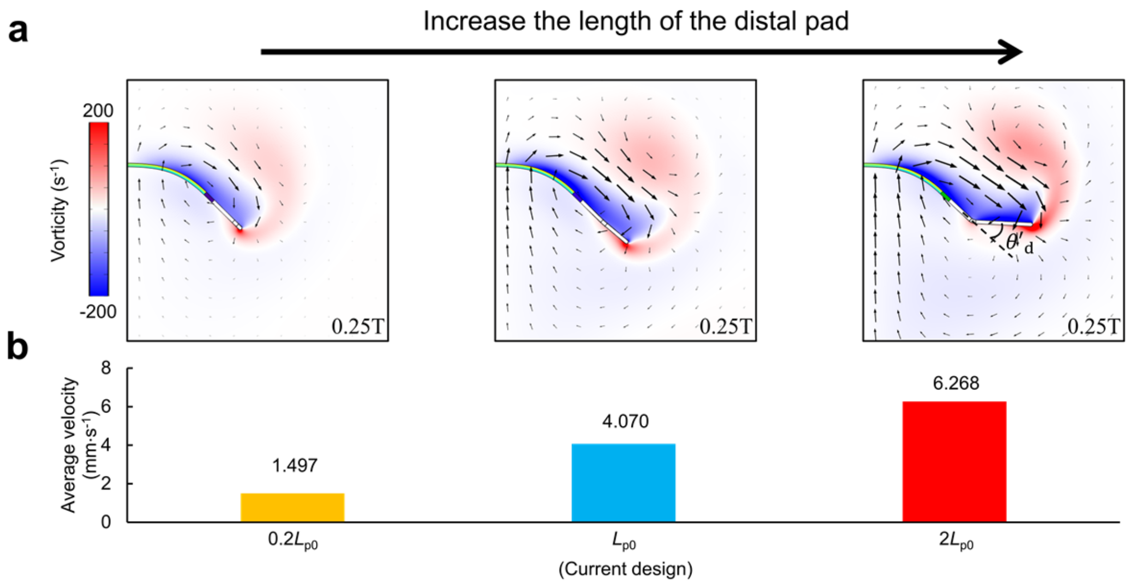

Supplementary Figure 15. The influence of changing the length of the distal pad. a, The influence of the distal pad length on deformation amplitude (θ'_{d}). **b**, The influence of the distal pad length on average swimming velocity.

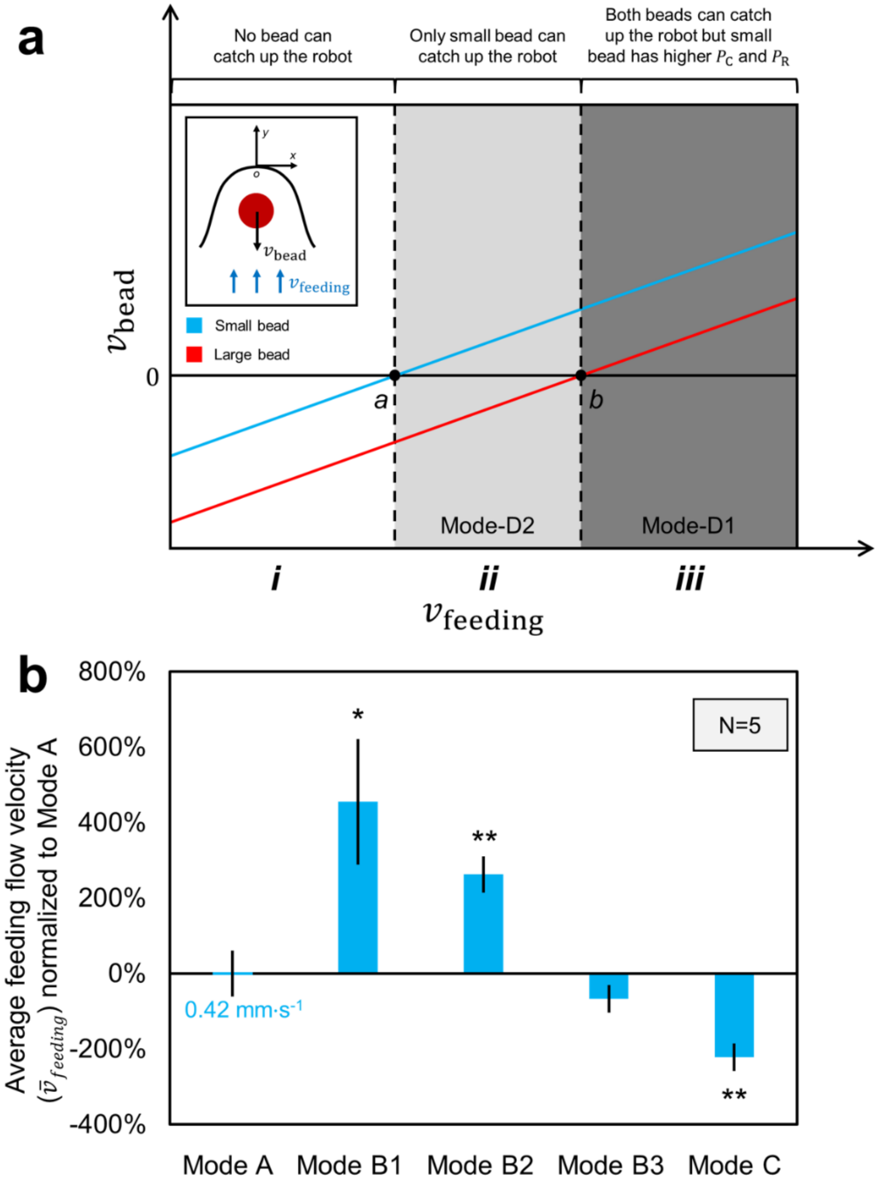

Supplementary Figure 16. Illustration of the mechanism for selectively transporting beads of two sizes. a, The blue and red lines indicate, respectively, the steady-state velocities of the small and the large beads being trapped by the upward feeding flow. Note that all velocities are expressed in the robot's body-attached reference frame (x, y, z). Therefore, a positive v_{bead} suggests the bead can catch up with the moving robot while a negative v_{bead} indicates the bead can escape through Mechanism-3. The black dashed lines passing through the point a and b separate the $v_{feeding}$ axis into three regions. The regions where the Mode D1 and Mode D2 work are shaded. It should be noted that the velocity profiles here are not plotted with specific values. Instead, they just illustrate the variation trend of v_{bead} respect to $v_{feeding}$. **b**, The average feeding flow velocity measured in five basic swimming modes. The error bars represent the standard error of the mean. N is the number of the trials.

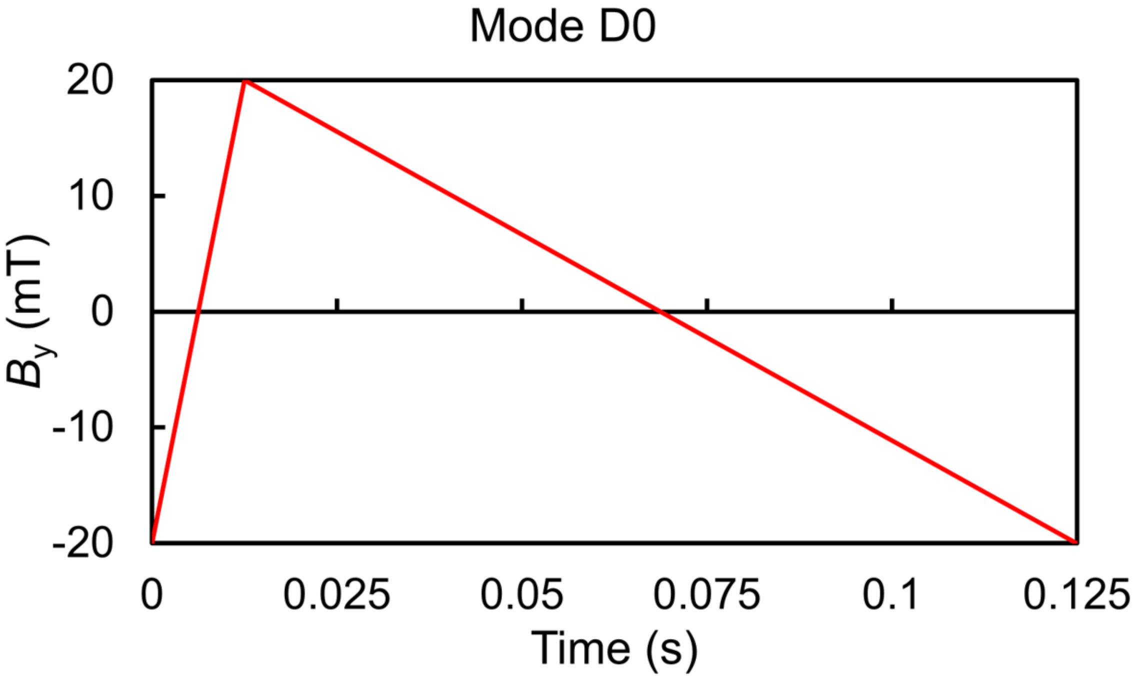

Supplementary Figure 17. Control signal for Mode D0.

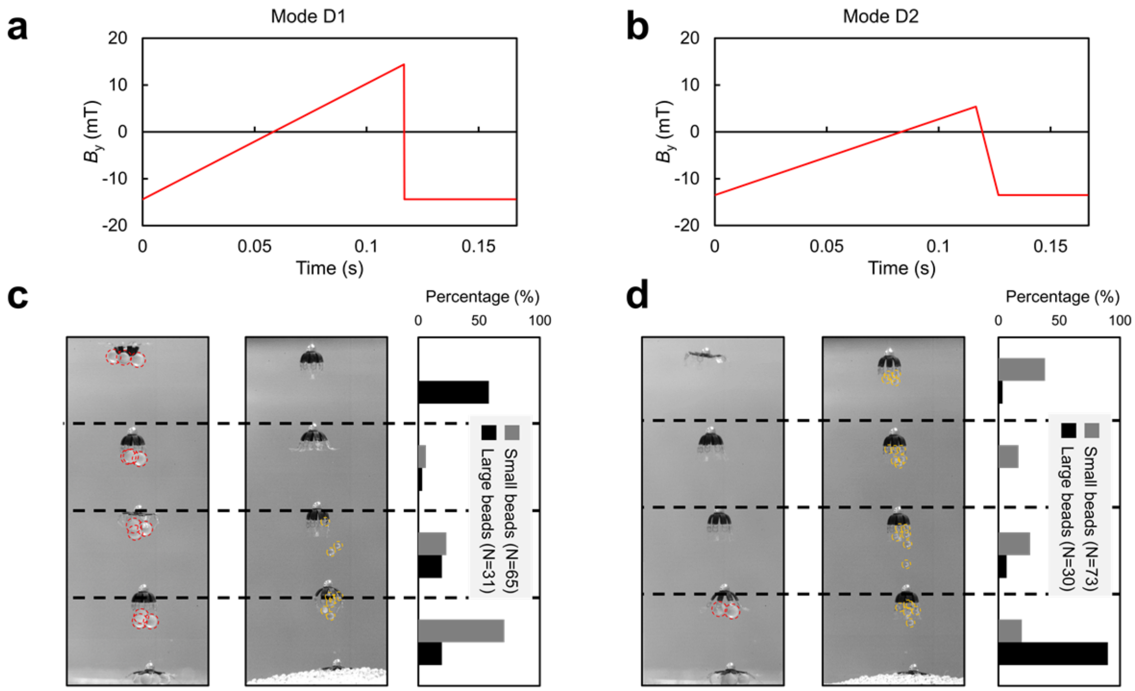

Supplementary Figure 18. Experimental investigation for selective transportation. a & b, Control signals of Mode D1 and Mode D2. **c & d**, The transportation performance of Mode D1 and Mode D2 regarding beads with large (left column) and small sizes (middle column). The beads escaping at each height interval (segmented into four zones by black dashed lines) are shown as the percentage (right column) of the overall beads being transported. Beads being transported out from the field of view are considered to escape in the fourth zone (uppermost). N is the number of the counted beads. See Supplementary Note 12 for more details.

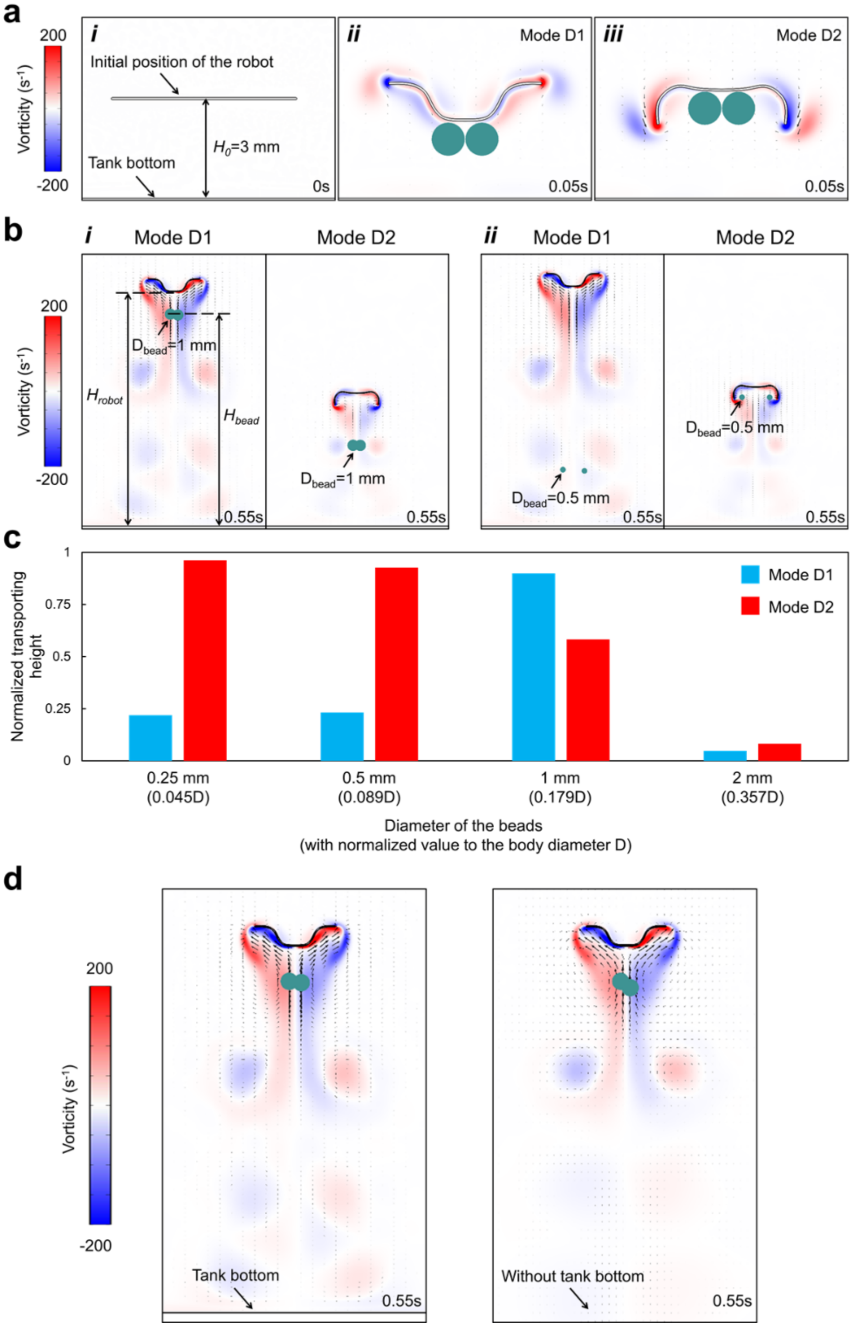

Supplementary Figure 19. Simulation for beads transportation. a, The virtual robot is modeled as a 2D beam and swims from the initial position that is 3 mm away from the tank bottom (0 s). The virtual beads are deployed under the robot at the end of the first recovery phase (0.05 s). **b**, The simulation results of large (D_{bead} = 1 mm) and small beads (D_{bead} = 0.5 mm) match the observations from the experiments. **c**, The normalized transporting heights achieved with different beads size and kinematics. **d**, The simulation on boundary effect (Tank bottom). The current result does not show significant impacts of the tank bottom on transportation performance.

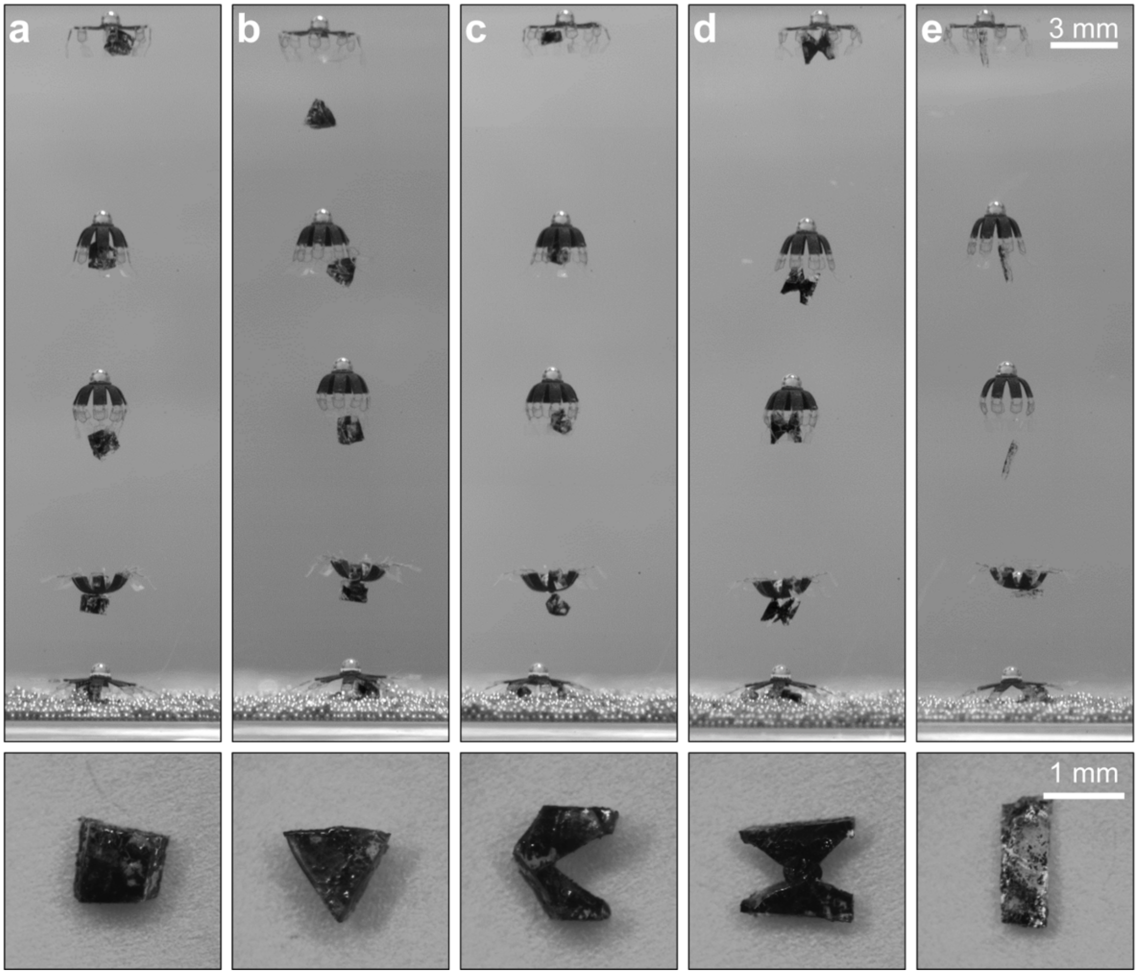

Supplementary Figure 20. Demonstration of transporting irregular objects. The objects used are made of PDMS and shown in the second row. Mode D1 is used throughout the experiments.

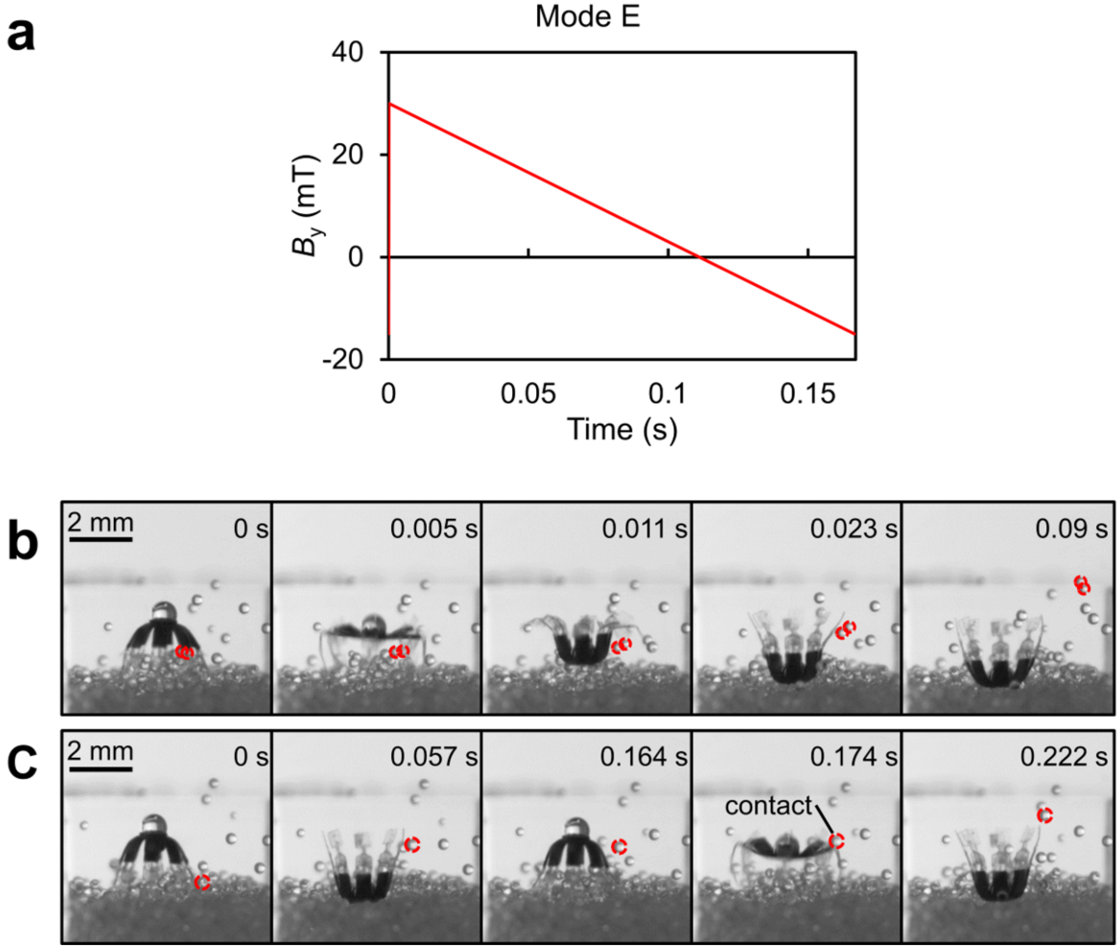

Supplementary Figure 21. Experimental investigation for burrowing. a, The control signal of Mode E. **b**, Beads are expelled by fluidic flow during recovery. **c**, Beads are expelled by fluidic flow in the first recovery phase (0 ~ 0.057s) and further beaten away by physical contact of the lappet in the second recovery phase (0.174 ~ 0.222s). See Supplementary Note 13 for more details.

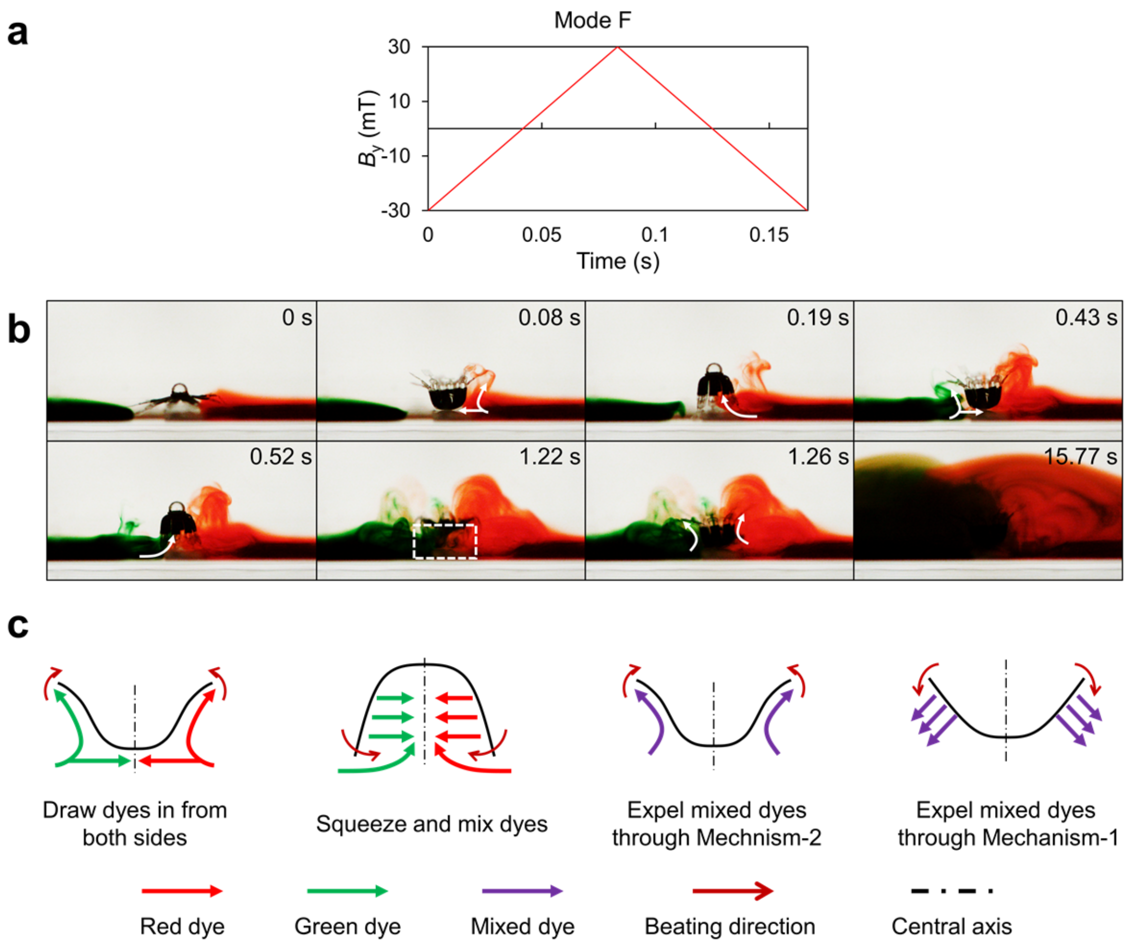

Supplementary Figure 22. Experimental investigation of local mixing. a, The control signal for Mode F. **b**, The motion sequence of local mixing. The white arrows indicate the transportation direction of the dyes. The white dashed box indicates the area in which the dyes are mixed. **c**, The illustration of the local mixing mechanism. See Supplementary Note 14 for more details.

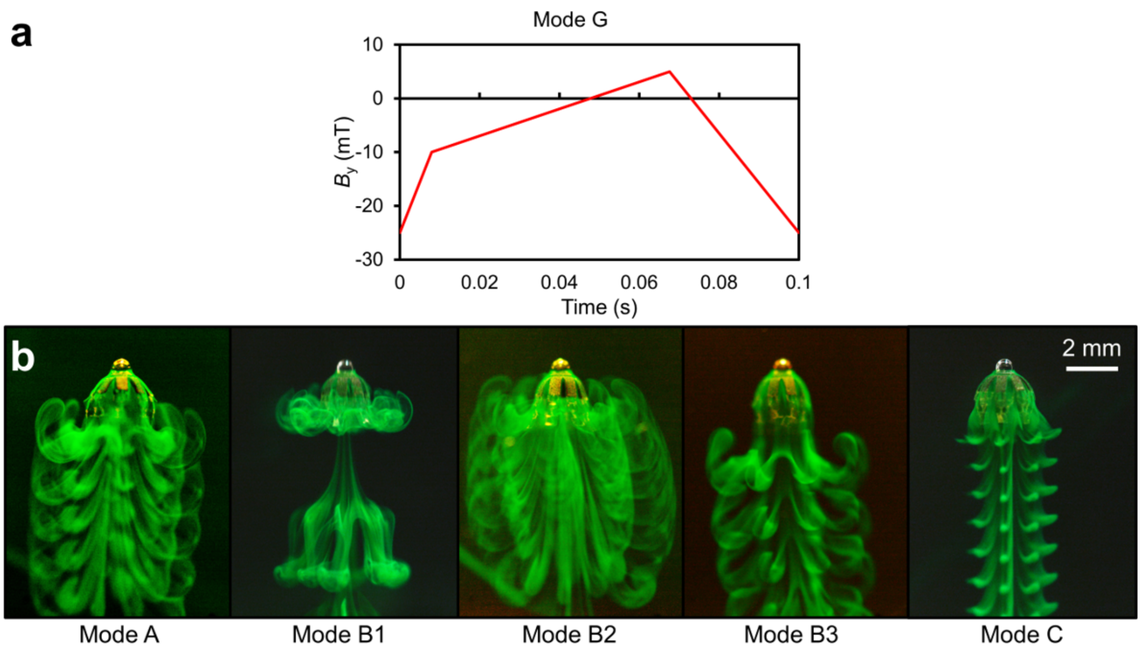

Supplementary Figure 23. Experimental investigation of generating a desired chemical path. a, The control signal for Mode G. **b**, The dye tree structures created by the five basic swimming modes. See Supplementary Movie 7 and Supplementary Note 15 for more details.

Table

**Estimating escaping probability (Methods)**	
P_{out}	The probability of a trapped bead being expelled
P_{c}	The probability of a trapped bead escaping through Mechanism-1 during contraction
P_{R}	The probability of a trapped bead escaping through Mechanism-2 during recovery
$E_{\text{retain-1}}$	The expectation of the retaining cycles of a trapped bead being expelled through Mechanism-1
$E_{\text{retain-2}}$	The expectation of the retaining cycles of a trapped bead being expelled through Mechanism-12
**The kinematic metrics (Supplementary Note 5)**	
F	The bell fineness
h	The bell height
D	The bell diameter
$\mathbf{u}_{\mathrm{l}}$	The instantaneous lappet velocity in body-attached frame
$\mathbf{X}_{\mathrm{ref}}(t)$	The coordinate of the reference point on lappet in the global frame
$\mathbf{X}_{\mathrm{bubble}}(t)$	The coordinate of the bubble in the global frame
Δt	The time interval between two frames
**The Reynolds number calculation (Supplementary Note 6)**	
Re_{B}	The Reynolds number of the flow around the body
Re_{L}	The Reynolds number of the flow around the lappet
$\mathbf{u}_{\mathrm{b}}$	The instantaneous velocity of the robot
d_{l}	The lappet width.
v	The kinematic viscosity of water
**The work done by magnetic torque (Supplementary Note 7)**	
E	The work done by magnetic torque in one cycle
T	The duration of one cycle
r_{core}	The radius of the magnetic composite elastomer core
θ_{e}	The angular deformation of the element
$\mathbf{R}(\theta_{\mathrm{e}})$	The rotation matrix of rotating angle θ
A	The cross-section area of one arm of the core
$\mathbf{m}$	The magnetization profile along the lappet of the magnetic core
$\mathbf{B}$	The external magnetic field
$\boldsymbol{\omega}$	The angular velocity of the element
d	The swimming distance per cycle
G_{robot}	The gravity of the robot
**The effective boundary coverage rate (Supplementary Note 8)**	
BC	The boundary coverage rate
δ	The thickness of the boundary layer between two adjacent lappets
Re_{local}	The local Reynolds number along the lappet
$\mathbf{X}$	An arbitrary point on lappet in the global frame
g	The gap distance between the adjacent lappets
r	The distance between the infinitesimal element and the central axis
r_{CD}	The radius of the central disk

α	Half of the angle between two adjacent lappets
r_{body}	The radius of the robot body
$A_{\text{actual\_central\_disk}}$	The area of the effective central disk
A_{outside}	The total area of the eight lappets outside the central disk
A_{total}	The area of the circumscribed circle of the robot body
**Metrics for object collecting performance (Supplementary Note 9)**	
Q_{exchange}	The exchange rate of the water volume
t_{R}	The duration of recovery
Vol_{feeding}	The volume of water sucked into the sub-umbrella region during recovery
v_{drift}	The velocity of the drift flow induced by the robot translational motion in the global frame
v_{robot}	The velocity of the robot in the global frame
$L(t)$	The length of the reference line
**Dynamic model for propulsion (Supplementary Note 10)**	
θ	The beating angle
h_{bell}	The height of the bell
r_{bell}	The radius of the projection of the bell
l_{total}	The sum of the radius of the magnetic elastomer core and the length of the passive lappet
$\mathbf{F}$	The resultant force acting on the robot
$\mathbf{T}$	The thrust generated by beating the lappet
$\mathbf{A}$	The virtual mass force
$\mathbf{G}$	The resultant force of the gravity and the buoyancy
m	The mass of the robot
$\mathbf{u}$	The propulsion velocity of the robot
c_{lappet}	The drag coefficient of the infinitesimal element along the lappet
ρ_{f}	The density of the water
c_{bell}	The drag coefficient of the robot body
S_{bell}	The projection area of the robot body
α	The added mass coefficient of the robot body
V	The volume of the sub-umbrella region
S_{C}	The translation distance of the robot during the contraction phase
S_{R}	The translation distance of the robot during the recovery phase
**Analyzing the mechanism of selective transportation (Supplementary Note 12)**	
F_{d}	The Stokes drag of the bead
G_{bead}	The gravity of the bead
F_{inertial}	The inertial force acting on the bead
η	The dynamic viscosity of the water
v_{bead}	The velocity of the bead falling against the upward drift flow
v_{feeding}	The feeding flow velocity at the time instant studied
$\overline{v}_{\text{feeding}}$	The average feeding flow velocity in one cycle
τ_{bead}	The relaxation time of the bead

Supplementary Table 1. Nomenclature

Supplementary Note 1. Kinematics of the jellyfish ephyra

The robot design is based on the kinematics of jellyfish ephyra. We extract the body profiles of a free-swimming jellyfish ephyra during one cycle from the supplementary video of Feitl *et al.*[1] In Supplementary Figure 4, two critical features can be observed. First, the central part of the body (marked with the black dashed box) can alternatively bend up and down. Second, the motion of the lappet (marked with the red dashed box) during the contraction phase is different from the recovery phase. During the contraction, the ephyra tends to enlarge the wetted area to enhance the thrust. During the recovery phase, however, the ephyra tends to decrease the wetted area to reduce the drag. To replicate such locomotion, the lappet of the robot is designed with two parts (Fig. 1a): the arm of the magnetic composite elastomer core that can be driven by the external magnetic field (**B** field), and the passive lappet that has different bending capabilities during the contraction and the recovery phase.

Supplementary Note 2. Coordinate systems

We use four reference frames in this paper. (i) The global frame (X, Y, Z) which is a stationary frame fixed in space (Supplementary Figure 3a). The average velocity of the robot, the velocity and the vorticity of the flow field are all calculated within this reference frame. (ii) The body-attached frame (x, y, z), whose origin fixes at the center top of the bubble, moves along with the robot (Supplementary Figure 16a). The external magnetic field (**B**) and the kinematic metrics are all expressed or calculated in this reference frame. (iii) The local material reference frame (l, m, n) deforms with the magnetic composite elastomer core (Supplementary Figure 1g). The calculation of the boundary layer is within this reference frame. (iv) The body frame (x', y', z') is a stationary frame[2]. Its axis direction is coincident with the body-attached frame at any instant while its origin always situates at the center of the bottom boundary of the sub-umbrella region (Fig. 3b). The exchange rate of the water volume and the average feeding flow velocity are all calculated in this frame.

Supplementary Note 3. Cut-off frequency and time constant of the Helmholtz coil system

The time constant τ of the coil system (Supplementary Figure 3a) used for characterization experiments should be short enough to let the coil respond to the rapid changing control signal. If the τ is too large, the magnitude of the output **B** field will be significantly smaller than the desired value and the robot lappet may beat too weakly to propel upwards. To determine the time constant, we first experimentally measure the cut-off frequency f_c of the Helmholtz coil system. The f_c of both the upper and lower electromagnets in the system are measured individually and the lowest value is recorded as the cut-off frequency of the whole coil system, which is 80 Hz. The τ of the coil system is then calculated with $\tau = \frac{1}{2\pi f_c}$, by which we obtain 0.002 s. It should be noted that the shortest phase designed for the five basic swimming modes lasts 0.03 s (the contraction phase of Mode B1), which is 15 times longer than τ. Therefore, we assume that the output magnetic field of the coil system can follow the desired signal pattern in all characterization experiments.

Supplementary Note 4. Adjustment to the control signals for compensating damping effects

Simply changing the corresponding phase of the control signal does not create desired kinematics. There are two reasons for this. Firstly, if the control signal changes too rapidly, the coil system may not be able to respond, introducing the loss in magnitude of the output magnetic field. However, as discussed in Supplementary Note 3, this is not the leading cause of the discrepancy here as we make sure the time constant of the coil, τ, is shorter in compared with the phase duration. Secondly, the damping force (*e.g.* the hydrodynamic force) acting on the robot body increases the energy dissipated in the fluid, which decreases the deformation amplitude of the robot body compared to the static case. The damping forces have a complex relation to the kinematics of the robot.

To compensate the decrease in lappet beating amplitude due to the damping force, we have to adjust the control signals by changing the amplitude or phase duration. For example, to achieve a higher ω_C, we first shorten the contraction duration, t_C, of the control signal of Mode A from 0.13 s to 0.01 s to prescribe the signal of Mode B1. However, t_R, θ_R, θ_C, θ_C - θ_R and ω_R all change slightly in the meantime due to the damping effects, which is undesired. To make these kinematic parameters unchanged compared to Mode A, the minimum value and the recovery phase duration of the control signal of Mode B1 are further adjusted to be, respectively, -17 mT and 0.29 s. In this way, the achieved t_R, θ_R, θ_C, θ_C - θ_R and ω_R do not see significant change (Fig. 2b, Supplementary Figures 5a-c). Similarly, the control signals of Mode B2, B3 and C are all adjusted in this way.

The experimentally measured kinematic parameters are used as the criterion for the above compensation. To experimentally measure the realized kinematic parameters, *i.e.*, t_R, t_C, θ_R, θ_C, $\theta_R - \theta_C$, ω_C, and ω_R, two key frames must be pinpointed from the videos: the frame in which the recovery phase just ends and the frame in which the contraction phase just ends. These two key frames are fully determined by the motion of the magnetic composite elastomer core. The time instant when the core beats up and reaches its maximal deformation during recovery is assumed to be the end time of the recovery phase. The time instant when the core beats down and reaches its maximum deformation during the contraction is assumed to be the end time of the contraction phase. Then, t_R and t_C are calculated by counting the number of the frames between these two key frames. At these two key frames, θ_R and θ_C are measured following the definition in the inset of Fig. 2b, and are used to calculate the beating amplitude ($\theta_R - \theta_C$) and the angular velocities (ω_C and ω_R). The results are shown in Fig. 2b and Supplementary Figures 5a-c. Through the signal adjustment, all of the control signals can achieve desired kinematics.

Supplementary Note 5. Calculation of the kinematic metrics

Two kinematic metrics, *i.e.*, the bell fineness and the lappet velocity, are used to demonstrate the similarity in kinematics between the robot swimming with Mode A and the jellyfish ephyra recorded in a previous research[1]. The bell fineness is defined with the following equation:

$$F(t) = \frac{h(t)}{D(t)}, \tag{1}$$

where $h(t)$ is the time-varying bell height and $D(t)$ is the time-varying bell diameter. h and D are measured directly from the high-speed image sequence. The lappet velocity $\mathbf{u}_\mathrm{l}$, which is defined in the robot's body-attached frame, is calculated with the following equation:

$$\mathbf{u}_\mathrm{l} = \frac{[\mathbf{X}_\mathrm{ref}(t)-\mathbf{X}_\mathrm{bubble}(t)]-[\mathbf{X}_\mathrm{ref}(t-\Delta t)-\mathbf{X}_\mathrm{bubble}(t-\Delta t)]}{\Delta t}, \tag{2}$$

where $\mathbf{X}_\mathrm{ref}(t)$ and $\mathbf{X}_\mathrm{bubble}(t)$ are the coordinates of the reference point (the distal joint which is marked with a red point in Fig. 1a) and the top of the bubble at time t in the global frame. The reference point has the same relative position to the body as rhopalia which is used to calculate the lappet velocity in animal[1]. The time interval Δt between two frames is 0.02 s, which is the same as that adopted in Feitl *et al.*[1]. The lappet velocities of both the robot and the animal are then normalized by the bell diameter measured when the body is at rest, which are 3 mm[1] and 5.6 mm, respectively.

Supplementary Note 6. Calculation of the Reynolds number

To demonstrate the similarity of the flow field between the robot swimming with Mode A and the animal, the Reynolds number of the flow around the whole body Re_B and the flow around the lappet Re_L are calculated following Feitl *et al.*[1]:

$$\begin{cases} Re_\mathrm{B} = \frac{\mathbf{u}_\mathrm{b}(t)D(t)}{\nu} \\ Re_\mathrm{L} = \frac{\mathbf{u}_\mathrm{l}(t)d_\mathrm{l}}{\nu} \end{cases}, \tag{3}$$

where $\mathbf{u}_\mathrm{b}(t)$ is the instantaneous body velocity at time t and is calculated with a time interval of 0.02 s, following that used in Feitl *et al.*[1]. $D(t)$ is the time-varying bell diameter. $\mathbf{u}_\mathrm{l}(t)$ is the lappet velocity defined is Supplementary Equation 2. d_l is the width of the lappet. ν is the kinematic viscosity of water at the temperature of 23 C°. When the robot swims with Mode A, Re_B varies from 7 to 95, and Re_L varies from 1 to 25. The Re_B matches the data from Feitl *et al.*[1], which reports that the *Aurelia aurita* ephyra, one typical scyphomedusae, swims in the fluid environment with $1 < Re_\mathrm{B} < 100$. In the same paper, Re_L of the ephyra ranges from 1 to 10, which is smaller than the robot. However, since Re_L in Feitl *et al.*[1] is measured from an ephyra with a smaller bell diameter (3 mm), a simple scaling to the result shows the Re_L of the robot is still within a reasonable range. The Reynold's number could be further fine-tuned by adjusting the design and control signal of the robot, based on the scientific question being investigated.

Supplementary Note 7. Work done by magnetic torque

The input energy is calculated by integrating the work done by magnetic torque on each infinitesimal element (Supplementary Figure 1g). We assume each arm of the magnetic core has the same magnetization profile and the same kinematics. The input energy for one

arm can then be multiplied by 8 to estimate the input energy of the whole body. The total magnetic work done by the external magnetic field is calculated with:

$$E = 8 \times \int_0^T \int_0^{r_{\text{core}}} A|(\mathbf{R}(\theta_{\text{e}})\mathbf{m}(l) \times \mathbf{B}(t)) \cdot \boldsymbol{\omega}(l,t)|dldt, \tag{4}$$

where $r_{\text{core}} = 1.5$ mm is the radius of the circumscribed circle of the core and is also assumed to be the length of the arm here. A is the cross section area of one arm of the magnetic core. $\mathbf{m}(l)$ is the volume magnetization ($A \cdot m^{-1}$) along one arm of the magnetic core (Supplementary Figure 1f). $\mathbf{R}(\theta_e)$ is a rotation matrix that accounts for the direction change of $\mathbf{m}(l)$ due to the bending of the core. $\mathbf{B}(t)$ is the external magnetic field given at time t and is assumed to follow the control signal waveform shown in Supplementary Figure 5d. $\boldsymbol{\omega}(l,t)$ is the angular velocity of the infinitesimal element at the position l, and it can be obtained by:

$$|\boldsymbol{\omega}(l,t)| = \left|\frac{\theta_{\text{e}}(l,t) - \theta_{\text{e}}(l,t-\Delta t)}{\Delta t}\right|, \tag{5}$$

where $\theta_e(l,t)$ can be measured from the high-speed video. With Supplementary Equations 4 and 5, the average input energies of Modes A, B1, B2, B3, and C can be calculated as 4.64×10^{-5} mJ, 7.92×10^{-5} mJ, 5.74×10^{-5} mJ, 4.75×10^{-5} mJ, and 1.90×10^{-5} mJ. The work done here is redistributed into four parts: (i) The strain energy stored inside the robot; (ii) The increase in kinetic energy of the robot; (iii) The increase in kinetic energy of the fluid; (iv) The heat losses. With E known, the COT of the robot can be simply calculated by:

$$\text{COT} = \frac{E}{G_{robot}d}, \tag{6}$$

where G_{robot} and d are, respectively, the gravity of the robot and the swimming distance per cycle.

When calculating E, we did not take the center hole out and it is included in Supplementary Equation 4. However, this only has a minor influence on the final results because this region produces negligible torque under the external **B** field. This is shown in Supplementary Figure 1f in which the magnetization profile has mainly M_y component within the region of the whole, suggesting a very small torque is produced under a **B** field changing along the y-axis. Therefore, we assume this does not negatively influence our calculation.

Supplementary Note 8. The contribution of the boundary layer to the boundary coverage rate

Here, we calculate the contribution of the boundary layer to the boundary coverage (BC) rate[1,3] of our robot. The boundary layer thickness along the lappet, δ, is a function of the local Reynolds number Re_{local} and the characteristic length[1], which can be expressed as:

$$\delta(l) = \frac{d_{\text{l}}}{\sqrt{Re_{\text{local}}}}, \tag{7}$$

where we take the width of the lappet d_l = 0.5 mm as the characteristic length. The Re_{local} is calculated by:

$$Re_{\mathrm{local}} = \left|\frac{\mathbf{U}_{\mathrm{l}}(l,t)d_{\mathrm{l}}}{v}\right|, \tag{8}$$

where $\mathbf{U}_l$ is velocity along the lappet. v is the kinematic viscosity of water at 23 C°. To obtain the velocity distribution $\mathbf{U}_l(l,t)$ along the lappet at the time instant t, the body profiles of the robot are extracted from the video. $\mathbf{U}_l(l,t)$ can then be simply calculated with:

$$\mathbf{U}_{\mathrm{l}}(l,t) = \frac{(\mathbf{X}(l,t)-\mathbf{X}_{\mathrm{bubble}}(t))-(\mathbf{X}(l,t-\Delta t)-\mathbf{X}_{\mathrm{bubble}}(t-\Delta t))}{\Delta t}, \tag{9}$$

where $\mathbf{X}(l,t)$ is the coordinate of an arbitrary point along the lappet in the global frame at time t. $\mathbf{X}_{bubble}(t)$ is the coordinate of the top point of the bubble in the global frame at time t. Δt = 0.02 s is the time interval between two frames. When calculating the boundary layer with Supplementary Equation 7, however, a problem rises. Since the local lappet velocity is very small at the proximal end of the lappet, the boundary layer at that region calculated by Supplementary Equation 7 achieves a very large thickness, overlapping the area of the magnetic core, which is unrealistic (Supplementary Figure 6a). To resolve this issue, only the boundary layer within the gap, *i.e.*, the valid boundary layer marked by the black dashed line in Supplementary Figure 6a, is considered. The valid boundary layer produced by the two adjacent lappets forms an extra area and enlarge the effective functional area of the original central disc, expanding the central disk radius from r_{CD} to r'_{CD} (Supplementary Figure 6b). The valid boundary layer depends on the time-varying gap distance $d_{gap}(l,t)$, which is defined in Supplementary Figure 6b, and can be calculated with:

$$d_{\mathrm{gap}}(l,t) \approx \begin{cases} 0 & (l < r_{\mathrm{CD}}\cos(\alpha)) \\ r(l,t)sin\alpha - d/2 & (l \geq r_{\mathrm{CD}}\cos(\alpha)) \end{cases}, \tag{10}$$

where $\alpha = \pi/8$. $r(l,t)$ (defined in Supplementary Figure 6c) measures the distance between the point (l,t) and the central axis of the robot. It can be directly measured from the video. When $l<r_{CD}cos(\alpha)$, $d_{gap} \equiv 0$ since the gap only exists outside the central disk (Supplementary Figure 6d). l_{border} is the position of the boundary border and satisfies the following condition:

$$d_{\mathrm{gap}}(l_{\mathrm{border}},t) = \delta_0(l_{\mathrm{border}},t), \tag{11}$$

where $\delta_0(l_{border},t)$ is the boundary layer thickness at the border of the effective central disk. We solve the Supplementary Equation 11 to obtain l_{border} at time t and then obtain $r'_{CD}(t)$ by:

$$r'_{\mathrm{CD}}(t) = \frac{l_{\mathrm{border}}}{cos\alpha}. \tag{12}$$

We calculate $r'_{CD}(t)$ at each time instant during one cycle and pick the minimum as the radius of the effective central disk of the whole period. Therefore, we can guarantee that the gap within the effective central disk can always be covered by the boundary layer. The average effective BC is estimated through the following equation:

$$\mathrm{BC} \approx \frac{A_{\text{actual central disk}}+A_{\text{outsides}}}{A_{\text{total}}} = \frac{\pi r'^{2}_{\mathrm{CD}}+nd_{\mathrm{l}}l_{\text{outsides}}}{\pi r^{2}_{\text{body}}}, \tag{13}$$

where $n = 8$ is the number of the lappets. A is the area of the corresponding part. $r_{\text{body}} = 2.8$ mm is the radius of the total bell. $l_{\text{outsides}} = r_{\text{body}} - r'_{CD}$ is the lappet length outside the effective central disk (Supplementary Figure 6d). With the existence of the valid boundary layer, the effective BC for five basic swimming modes (Modes A, B1, B2, B3, and C) are increased from 40.56% to, respectively, 45.36%, 41.51%, 43.15%, 43.88%, and 46.54%. Such an increase in BC can improve both the propulsion[3] and the object manipulation, as the extra boundary layer prevents the objects leaking through the inter-lappet gap from inside to outside the sub-umbrella region.

However, the effective BC rate estimated on our robot is still smaller than the real ephyra. The BC rate of the *Aurelia aurita* ephyra with a bell diameter of 5 mm varies from 50% to 80% according to previous biological studies[1,3]. There are two ways to improve the BC rate. In the first way, the lappet could be designed as trapezoid shape like that in Nawroth et al.[4] to make the umbrella shape of the robot more hydrodynamic continuous, if the sole purpose is to improve the performance of the robot on propulsion and object manipulation. However, the larger distal end of the lappet would restrict the maximal contraction of the robot and deviate the robot from the morphological feature of the real ephyra. Second, we can enlarge the central disc and the lappet width in an allometric way like that in real ephyra[1]. In this case, soft materials have to be fabricated between the inter-lappet gaps. As a first attempt, a new design with larger BC rate (60.87%, without considering the boundary layer) is realized by decreasing the overall size (with diameter of 4.6 mm), increasing the width of the distal and proximal pads (0.8 mm), and filling the gaps between the lappets with soft silicone rubber (Ecoflex 00-10). The newly-designed robot is shown in Supplementary Figure 7. The kinematics of the robot is prescribed based on the kinematics data of an ephyra with a diameter of 4.6 mm calculated from the fitting models proposed in Nagata et al.[5]. The beating amplitude of the robot is set to be 84.55 deg, which locates within the range of the biological observations (82.3 ~ 101.52 deg)[5]. The average bell fineness is set to be 0.46, which also matches the biological measurements (0.2 ~ 0.5)[5]. The resultant average swimming velocity of the robot is 2.11 mm s^{-1}, which exactly matches the velocity of a swimming ephyra with a diameter of 4.6 mm (2.05 ~ 2.91 mm·s^{-1})[5]. Due to the interference of the material between the gaps, the deformation of the magnetic composite elastomer core is not as good as the design we adopt in the main text. In the future, foldable soft materials such as gelatin[6] can be used as the inter-lappet material.

Supplementary Note 9. PIV data analysis

General PIV results. The sequence of the velocity and vorticity fields of the five basic swimming modes are shown in Supplementary Figure 8. In general, all these swimming modes have a similar vortex evolution pattern which is comparable to an ephyra[4]. At the beginning of the contraction phase, the starting vortex is observed to form at the distal end of the lappet (red dashed circles at 0T of Mode A in Supplementary Figure 8a). During the contraction phase, the stopping vortex also gradually forms at the top surface of the lappet

(green dashed circles at 0.21T of Mode A in Supplementary Figure 8a). At the end of the contraction, the starting vortex dissipates and the stopping vortex still attaches to the lappet (green dashed circles at 0.36T of Mode A in Supplementary Figure 8a). During the recovery, the stopping vortex gradually sheds from the robot body, and the vorticity magnitude gradually decreases (green dashed circles at 0.50T and 0.70T of Mode A in Supplementary Figure 8a).

The Mode B1 is an exception to the flow structure described above. At the end of the contraction, the starting vortex of Mode B1 is still significant (at 0.13T of Mode B1 in Supplementary Figure 8a). The stopping vortex splits into two parts. One still attaches to the lappet, and another one departs from the body. This proportion of the stopping vortex and the starting vortex form a vortex pair, drifting away quickly downwards (Marked with brown dashed circles at 0.13T and 0.33T of Mode B1 in Supplementary Figure 8a). This is one important contributor to the decreasing retaining cycles shown in Fig. 4b.

The strength of the stopping vortex, measured by circulation, is also shown in Supplementary Figure 8b. The circulation is a metric for evaluating the magnitude and the scale of the vortex[7]. For each mode, 12 to 26 vortexes are measured at the time instant when the magnetic core recovers to be flat (*e.g.*, at 0.70T of Mode A in Supplementary Figure 8a). At this time instant, the stopping vortex fully sheds from the body and hence can be easily pinpointed. With a stronger contraction or recovery phase (Mode B1 and Mode B2), the strength of the stopping vortex is significantly enhanced. With a smaller amplitude (Mode C), the strength of the stopping vortex is reduced. The strength of the stopping vortex of Mode B3 is weaker than Mode A, which could be due to the energy dissipation during the glide phase. At last, it has to be noted that stronger stopping vortex also indicates stronger feeding flow[4]. So the relative strength of each mode on this graph agrees with that of the Q_{exchange} (Fig. 3c) and $\overline{v}_{\mathrm{feeding}}$ (Supplementary Figure 16b).

At last, it has to be noted that the current study only focuses on the flow field right under the lappets as it is the main contributor to the propulsion and object manipulation in our experiment. Moreover, the flow field between the adjacent lappets (inter-lappet area) is not investigated yet and is assumed to be similar to that under the lappet. However, the difference does exist between these two areas. Further investigation will be carried out in the future to reveal its effect on different performance indices.

Exchange rate of the water volume. Defined in Fig. 3b, the exchange rate of the water volume, Q_{exchange}, is estimated based on the 2D velocity field obtained by PIV experiments. It quantifies how much water can be exchanged into the sub-umbrella region within one cycle, through the bottom boundary of the sub-umbrella region. The black dashed reference line in Supplementary Figure 9a is used to indicate the lower boundary of the sub-umbrella region. The reference line is either the connection between two lappet tips or the projection of the robot body. The reader can also refer to Supplementary Figure 3b for a more detailed definition of the sub-umbrella region. The frame used here for calculation has its origin, o', positioned at the middle point of the reference line.

Supplementary Figure 9b shows v_{robot} is larger than $\overline{v}_{\text{drift}}$ (the average of $\overline{v}_{\text{drift}}$ along the reference line) during the contraction while is smaller during the recovery. This indicates that the water is sucked in through the bottom boundary during the recovery phase and expelled during the contraction phase. We calculate the volume of the feeding flow (Vol_{feeding}) sucked in during the recovery to estimate the flow volume exchanged during one cycle. If we assume the feeding flow has an axisymmetric structure in 3D space, Vol_{feeding} can be expressed as:

$$Vol_{\text{feeding}} = 2\pi \int_0^{t_{\text{R}}} \int_0^{L(t)/2} x'\left(v_{\text{drift}}(x',t) - v_{\text{robot}}(t)\right) dx'dt, \tag{14}$$

where $L(t)$ is the length of the reference line. t_{R} is the duration of the recovery. v_{drift} and v_{robot} are the velocities of the drift flow and the robot, respectively. Vol_{feeding} of the trials from which Supplementary Figure 9b is obtained are, separately, 38.13 mm^3, 69.32 mm^3, 44.41 mm^3, 35.94 mm^3 and 20.85 mm^3 (Modes A, B1, B2, B3, and C). We also perform the integration within one complete cycle (that is, t_{R} in Supplementary Equation 14 is replaced by T), and the results of the same trials are, separately, 5.20mm^3, 17.71 mm^3, -5.91 mm^3, 0.42 mm^3 and 0.87 mm^3 (Modes A, B1, B2, B3, and C). The integration values within one complete cycle are more than three times smaller than the integration within the recovery phase. Therefore, the current discussion assumes the water volume sucked in through the bottom line during the recovery phase is the same as the water volume expelled through the bottom line during the contraction phase. However, the Vol_{feeding} is affected by many other factors, *e.g.* the flow leaks through the inter-lappet region. Therefore, more analyses will be carried out in the future.

With the above assumption, Q_{exchange} can then be calculated by:

$$Q_{\text{exchange}} = \frac{Vol_{\text{feeding}}}{T}, \tag{15}$$

where T is the duration of one cycle. From Supplementary Equation 14, both $v_{\text{drift}}(x', t)$ and $v_{\text{robot}}(t)$ influence the Q_{exchange}. This can also be shown in Supplementary Figure 9b, in which the area enclosed by two velocity profiles indicates the amount of water being exchanged. Because Vol_{feeding} is integrated within the recovery, the following discussions do not incorporate the contraction phase.

In Mode B1, a stronger contraction induces the highest drift flow velocity (The blue line of Mode B1 moves upwards relative to Mode A in Supplementary Figure 9b. Note that the range of the *y*-axis tick for Mode B1 is larger than that for Mode A). Although the swimming velocity of the robot also increases (the red profile of Mode B1 moves upwards relative to Mode A in Supplementary Figure 9b), the area between two profiles is still enlarged, indicating the water collected by Mode B1 is increased.

In Mode B2, a stronger recovery makes the robot move downwards more rapidly (the red line of Mode B2 moves downwards relative to Mode A in Supplementary Figure 9b), enlarging the enclosed area, which helps to increase the flow volume entering the sub-umbrella region.

In Mode B3, there is no fluid exchange during gliding (indicated by the overlapping of the red and the blue lines within the glide phase as shown in Supplementary Figure 9b). Therefore, no collecting happens within the glide phase, which decreases the overall object collecting speed. This might be a reason why the ephyra does not possess gliding behavior as it makes it slower to capture prey.

In Mode C, both the drift flow and the robot downward motion are decreased (the red and blue lines of Mode C, respectively, move up and down relative to Mode A in Supplementary Figure 9b), greatly reducing the amount of water sucked into the sub-umbrella region.

Supplementary Note 10. Discussions on propulsion performance by a dynamic model
As the first step to model the dynamics of the robot, here we investigate the impact of changing the kinematic parameters (t_C, t_R, t_G, and θ_R) on propulsion performance through a dynamic model. For simplification, we assume each lappet of the robot is rigid and rotates relative to a revolution joint at the proximal end of the lappet during beating (Supplementary Figure 10a). The lappet is further represented as a linear array of infinitesimal elliptical cylinder elements following Nawroth *et al.*[4]. The beating angle of the lappet, θ, is modeled using the following formula:

$$\begin{cases} \theta = \frac{1}{2}(\theta_R - \theta_C)\left(\cos\left(\frac{\pi}{t_C}t\right) + 1\right) + \theta_C & (0 \le t \le t_C) \\ \theta = \theta_C & (t_C < t \le t_C + t_G) \\ \theta = \frac{1}{2}(\theta_R - \theta_C)\left(-\cos\left(\frac{\pi}{t_R}(t - t_C - t_G)\right) + 1\right) + \theta_C & (t_C + t_G < t \le t_C + t_G + t_R) \end{cases}, \tag{16}$$

where θ_C and θ_R are, respectively, the contraction and recovery angles. t_C, t_R, and t_G are, respectively, the durations of the contraction, recovery and glide phases. The angular velocity of each phase can then be obtained by taking the derivative of the Supplementary Equation 16:

$$\boldsymbol{\omega} = \dot{\theta}. \tag{17}$$

We then can estimate the speed of each infinitesimal element at location l to be $v_{\text{element}} = l\omega$. The height of the bell, h_{bell}, and the radius of the bell, r_{bell}, are time-varying and can be calculated with the following equations:

$$\begin{cases} h_{\text{bell}} = l_{\text{total}}|sin\theta| \\ r_{\text{bell}} = l_{\text{total}}|cos\theta| \end{cases}, \tag{18}$$

where l_{total} is the sum of the radius of the magnetic elastomer core and the length of the passive lappet. Due to the design of the passive lappet, l_{total} is assumed to be different in contraction and recovery phases. From the kinematics of Mode A, we measure that l_{total} = 2.2 mm during the contraction and l_{total} = 2 mm during the recovery.

The resultant force **F** accelerating the robot is modeled as the sum of the thrust force **T**, the drag force **D**, the virtual mass force **A** and the gravity **G**[8]:

$$\mathbf{F} = \mathbf{T} - \mathbf{D} - \mathbf{A} - \mathbf{G} = m\frac{d\mathbf{u}}{dt}, \tag{19}$$

where m and **u** are the mass and velocity of the robot, respectively. The paddling propulsion is drag-based propulsion which relies on the drag force produced during beating to generate thrust[9]. Therefore, the thrust force **T** is calculated by integrating the drag force acting on each infinitesimal element of the eight lappets:

$$\mathbf{T} = -8 \times \int_0^{l_{\text{total}}} \frac{1}{2} c_{\text{lappet}} \rho_{\text{f}} d_{\text{l}} v_{\text{element}} \boldsymbol{v}_{\text{element}} dl, \tag{20}$$

where ρ_{f} is the water density, d_{l} is the width of the lappet, $\boldsymbol{\omega}$ is the angular velocity. c_{lappet} is the drag coefficient of each infinitesimal elliptical cylindrical element. c_{lappet} is a function of the local Reynolds number along the lappet, Re_{local} (see Supplementary Note 6), and is estimated based on the data provided in Masliyah *et al.*[10]. The drag **D** acting on the body is calculated using the following equation:

$$\mathbf{D} = \frac{1}{2} c_{\text{bell}} \rho_{\text{f}} S_{\text{bell}} u \mathbf{u}, \tag{21}$$

where $S_{\text{bell}} = \pi r_{\text{bell}}^2$ is the projection area of the body and c_{bell} is the drag coefficient of the body which is represented as a circular disk translating normal to the flow. In the moderate Reynolds number range, the drag coefficient of the circular disk is close to the drag coefficient of a sphere[11], which can be fitted with the following equation[12]:

$$c_{\text{bell}} = \frac{24}{Re_{\text{B}}}\left(1 + 0.173 {Re_{\text{B}}}^{0.657}\right) + \frac{0.413}{1 + 163000 {Re_{\text{B}}}^{-1.09}}. \tag{22}$$

The virtual mass force **A** is caused by accelerating the water surrounding the body. It is calculated with the following equation:

$$\mathbf{A} = \alpha \rho_{\text{f}} V \frac{d\mathbf{u}}{dt}, \tag{23}$$

where α and V are the added mass coefficient and the volume of the sub-umbrella cavity, respectively[8]:

$$\begin{cases} \alpha = \left(\frac{h_{\text{bell}}}{r_{\text{bell}}}\right)^{1.4} \\ V = \frac{2\pi h_{\text{bell}} r_{\text{bell}}^2}{3} \end{cases}. \tag{24}$$

With the above formulation, the velocity of the robot, **u**, is solved numerically by implementing the Runge-Kutta method with variable time steps in Matlab (version 2018a, Mathworks). The calculation is conducted for eight complete cycles, and the last five cycles are used to calculate the average velocity of the robot, $\bar{v}_{\text{robot}}$. This is to make sure the calculation of $\bar{v}_{\text{robot}}$ does not include the transition phase within the first several cycles. During the transition phase, the robot velocity is different from that achieved when the robot swims steadily. This may be because the robot is still accelerating during the first

few cycles and should be further investigated. Using the t_C, t_R, θ_C and θ_R measured in Supplementary Note 4, the predicted $\overline{v}_{robot}$ of Modes A, B1, B2, B3 and C are 4.26 mm·s^{-1}, 26.07 mm·s^{-1}, 4.20 mm·s^{-1}, 4.70 mm·s^{-1} and 0.01 mm·s^{-1}. The $\overline{v}_{robot}$ of these five modes measured from experiments are 3.95 mm·s^{-1}, 16.13 mm·s^{-1}, 4.16 mm·s^{-1}, 4.39 mm·s^{-1} and 1.72 mm·s^{-1}. The biggest discrepancy exists at Mode B1 and Mode C. This may be because the simplified model doesn't incorporate the fluid-structure interaction between the surrounding flow and the passive lappets (Supplementary Note 11), which leads to a complex influence on the thrust and drag force acting on the robot. We will improve this model by incorporating the fluid-structure interaction in the future.

To investigate the impact of the duration of each phase on propulsion performance, we vary t_C, t_R, t_G, and θ_R while keeping other kinematic parameters unchanged to calculate $\overline{v}_{robot}$. The results are shown in Supplementary Figures 10b-g. Overall, the variation of the velocity predicted by the model matches that measured by experiments.

We vary t_C in Supplementary Figure 10b. Specifically, we keep t_R = 0.27 s, t_G = 0, θ_C = 0.6 rad, θ_R = 2.2 rad, which are the same as Mode A, and vary t_C from 0.01 s to 2 s. As expected, the relationship $\overline{v}_{robot} \propto \frac{1}{t_C}$ holds for the whole time range. In other words, faster contraction leads to higher swimming velocity.

We vary θ_R in Supplementary Figure 10c. Specifically, we keep t_C = 0.13s, t_R = 0.27 s, t_G = 0 s, θ_C = 0.6 rad, which are the same as Mode A, and vary θ_R from 0.6 rad to 2.7 rad. There should exist an optimal value for the beating amplitude to induce the highest swimming velocity. If the beating amplitude is too small, the thrust produced during contraction decreases. While if the beating amplitude is too large, the drag produced during recovery increases. Both of these effects can slow down the robot.

We vary t_R in Supplementary Figure 10d. Specifically, we keep t_C = 0.13s, t_G = 0 s, θ_C = 0.6 rad, θ_R = 2.2 rad, which are the same as Mode A, and vary t_R from 0.01 s to 2 s. If we decrease t_R of Mode A, the robot can first reach a velocity peak since the robot beats more frequently. However, if we continue to decrease t_R, the velocity of the robot can rapidly decrease, making the robot unable to swim upwards (negative velocity). This is because the recovery is so strong that it decelerates the robot so rapidly during the recovery, making the downward displacement in recovery even greater than the upward displacement in contraction. This can be supported by Supplementary Figure 10e, in which we plot the difference between the upward displacement during contraction, S_C, and the downward displacement during recovery, S_R:

$$\Delta S = S_C - S_R. \tag{25}$$

Besides decreasing the t_R, it should be noted that the velocity of the robot can gradually decrease and eventually becomes negative if t_R increases. This is because the recovery phase is so long that the robot starts to drop during the recovery. Since the velocity of the robot decreases much more rapidly by decreasing t_R than by increasing t_R, we choose to

deprive the swimming ability of the robot to realize functions at the tank bottom by greatly enhancing the recovery (burrowing and local mixing, see Supplementary Notes 13 and 14).

We vary t_G in Supplementary Figure 10f. Specifically, we keep t_C = 0.13 s, t_R = 0.27 s, θ_C = 0.6 rad and θ_R = 2.2 rad, which are the same as Mode A, and vary t_G from 0.01 s to 2 s. If we increase t_G of Mode A, the robot can first reach a velocity peak. This is because the robot maintains high velocity obtained at the end of the contraction phase during the gliding. Consequently, this increases the traveling distance per cycle and $\overline{v}_{robot}$. However, the velocity of the robot eventually reduces to negative if t_G continue increasing. This is because the gliding phase is so long that the robot starts to drop during the glide due to gravity. This is supported by the data shown in Supplementary Figure 10g.

Supplementary Note 11. Discussions on the design mechanism of the robot

The current design of the robot used in the main text is to particularly study the interaction between the robot body and the incurred flow structures, especially its application on the predation-inspired object manipulation capability. It is not an optimized design regarding various robotic tasks. As a first step towards optimizing the robot on different performance, studying the influence of changing design parameters is meaningful. In the following, we first discuss the influence of changing the thickness, Young's modulus and the magnetization of the magnetic composite elastomer core on its deformation. We then discuss the influence of changing the stiffness of the flexible joints and the length of the pads on kinematics and swimming velocity.

The influence of changing the design parameters of the magnetic composite elastomer core. The properties of the magnetic soft material used to build the magnetic composite elastomer core can greatly influence its deformation under the external magnetic field. Here we particularly investigate the influence of changing the thickness, Young's modulus and the magnetization magnitude of the material on core deformation at static condition. For simplification, the bending of the core is modeled as the deformation of a 2D beam (Supplementary Figure 1g). Therefore, the following equilibrium equation holds[13]:

$$[0\ 0\ 1]([\mathbf{R}(l)\mathbf{m}(l)] \times B) = -c\frac{EI}{wh}\frac{\partial^2\theta_e(l)}{\partial l^2}, \quad (26)$$

where $\mathbf{m}$ is the magnetization profile along the beam (Supplementary Figure 1f). $\mathbf{R}$ is the rotational matrix of each infinitesimal element. $\mathbf{B}$ is the external magnetic field. θ_e is the rotation deflection along the beam. w, h, E and I are the width, thickness, Young's modulus and the second moment of area of the beam. For the robot used in this paper, w, h, E and I are, $0.5\ mm$, $65\ \mu m$, $8.45 \times 10^4\ Pa$, and $1.1443 \times 10^{-17}\ m^4$. c is a scaling factor that is calibrated through experiments to compensate the discrepancy between the experiments and the numerical calculation. The body profiles obtained at a series of external magnetic fields ranging from -10mT to 8mT are used as the baseline to adjust c to make the predicted profiles match the observed results ($c = 10$). Although the 2D beam model is a simplified estimation to the real case, it can still capture the trend of the

deformation of magnetic composite elastomer core (Supplementary Figure 11a). After c has been determined, we systematically scale h, E, and the $\mathbf{m}$ magnitude to compare the beating angles of the core, θ_{core} (see the inset of Supplementary Figure 11b for the definition), achieved at different circumstances. The results are shown in Supplementary Figure 11b. In summary, the core deformation becomes less if the material becomes thicker, stiffer, or has lower magnetization.

The current model simplifies the 3D mechanical structure of the robot into a beam. This could be one potential reason why a scaling factor, c, exists. In the future, a more accurate representation of the robot, *e.g.* shell, plate or FEA model[14], will be investigated.

The influence of changing the design parameters of the passive lappets. Finite element analysis is conducted using the commercial software COMSOL Multiphysics 5.3a (COMSOL, Inc., Stockholm, Sweden) to investigate the deformation of the passive lappets and its impact on the swimming velocity. The kinematics of the passive lappets is dictated by its complex interaction with the surrounding fluid. In our analysis, the robot is modeled as a 2D beam composed of four components: the active part representing the deformation of the magnetic composite elastomer core, the directional proximal joint that can only passively bend during the recovery, the passive distal joint that can passively bend during both contraction and recovery, and the rigid distal and proximal pads (Supplementary Figure 12a).

For a fair comparison, the kinematics of the magnetic core is enforced to replicate that extracted from the experiments of Mode A. The distal and proximal pads are modeled as rigid bodies that cannot deform. The flexible proximal and distal joints are modeled with Neo-Hookean material model[15]. A damping term is added to the material model to reduce the oscillation and improve the convergence of the time-dependent solver. To reduce the computational cost, only half of the body is simulated (Supplementary Figure 12a). The unsteady, incompressible, symmetrical (symmetric boundary condition is applied along the symmetric axis shown in Supplementary Figure 12a), 2D Navier–Stokes equation is solved to obtain the hydrodynamic force, which in turn pushes the body to move. For the simulation in this section, the virtual robot swims far away from the computational boundary. Therefore, the boundary effects are considered to have no effects on the results.

We first investigate the influence of tuning the joint stiffness. In the simulation, the thickness of the flexible joint is set to be the same as the active magnetic core to avoid the sharp corners that may cause numerical instability. The width of the flexible joint is also fixed through all the cases for the ease of performing simulation in 2D. Therefore, the flexibility of the joints are tuned by only changing Young's modulus of the materials (E_{p} and E_{d}) that compose the proximal and distal joints. We first tune E_{p} and E_{d} to be E_{p0} and E_{d0} to make the achieved kinematics match that of Mode A from experiments (see the comparison of the kinematics between the experiment and the simulation in Supplementary Figure 12b). From this baseline, we then tune E_{p} and E_{d} to be higher or lower values to examine the influence of the joint stiffness on structure deformation and swimming velocity.

If the proximal joint becomes softer ($0.8E_{\mathrm{p0}}$), the maximal bending angle, θ_{p} (defined in Supplementary Figure 12c), and the average swimming velocity doesn't see significant change (Supplementary Figures 12c and 12d). This is because E_{p0} is observed to have almost minimized the wetted area of the robot (The projection area on the bottom plane) during the recovery. Further reducing E_{p0} doesn't significantly change the drag during the recovery. In contrast, the stiffer ($2E_{\mathrm{p0}}$ and $500E_{\mathrm{p0}}$) proximal joint leads to a smaller θ_p and reduces the average velocity (Supplementary Figures 12c and 12d). This is because the wetted area during the recovery increases the drag and slows down the robot.

In compared to changing the stiffness of the proximal joint, changing the stiffness of the distal joint has less influence on swimming velocity (Supplementary Figure 13b), although the bending angle, θ_{d}, does see a significant variation (Supplementary Figure 13a). According to the simulation, there should exist an optimal distal joint stiffness that maximize the swimming velocity, and our current design is close to this optimal value (Supplementary Figure 13b). This reflects a trade-off in choosing the stiffness of the distal joint. If the distal joint is softer, it bends more during the contraction and less thrust force is generated. If the distal joint is more rigid, it bends less during the recovery and larger drag force is introduced.

We then investigate the influence of tuning the pad length. To investigate this topic, we first set the Young's modulus of the proximal and distal joints, E_{p} and E_{d}, to be the same as the current robot design ($E_{\mathrm{p}} = E_{\mathrm{p0}}$, $E_{\mathrm{d}} = E_{\mathrm{d0}}$), and then tune the lengths of the proximal and distal pads, L_{p} and L_{d}, to be longer or shorter to examine their influences on the deformation of the passive lappets and the swimming velocity.

If we decrease L_{p} to be $0.2L_{\mathrm{p}}$, the bending angle, θ'_{p}(defined in Supplementary Figure 14a), becomes smaller. The simulation shows that the vortex shed from the body becomes weaker (Supplementary Figure 14a), and less thrust is generated during the contraction, resulting in lower swimming velocity (Supplementary Figure 14b). On the contrary, if we increase L_{d} to be $2L_{\mathrm{d}}$, the robot can produce higher thrust and acquire higher swimming velocity. This is because increasing L_{p} enlarges the wetted area during the contraction, while maintaining the wetted during the recovery to be the same. Therefore, higher thrust can be obtained. Similar results can also be obtained by varying the length of the distal pad (Supplementary Figure 15).

Although elongating the passive pad improves the propulsion, the choice of the pad length is restricted by the application of the robot. Additionally, continuously increasing the pad length will eventually make the magnetic core be not able to drive the passive lappet, reducing the propulsion velocity.

Supplementary Note 12. Discussions on selective transportation of beads with different sizes

The mechanism of realizing selective transportation. By only changing the kinematics, the robot can selectively transport beads with two different sizes. Polystyrene beads

(Polysciences, Inc.) with an average diameter of 550 µm (small beads) and 1 mm (large beads) and a density of 1.05 g·cc^{-1} are used for demonstration. These beads can mimic many micro-plastics scattered in the ocean[16].

For these heavier beads, they may fall out of the sub-umbrella region due to the gravity, in addition to the Mechanism-1 and 2 discussed in Fig. 4c. This is classified as Mechanism-3. To illustrate this concept, we can build a simplified model as shown in the inset of Supplementary Figure 16a in which a bead is inside the feeding flow produced by a moving robot. For simplification, we assume the robot is moving upwards with a velocity v_{robot} and acceleration a_{robot} in the global frame at the time instant we study (note we regard the velocity and acceleration of the body-attached frame (o, x, y, z) the same as that of the robot). The feeding flow velocity v_{feeding} and bead velocity v_{bead} are expressed in the body-attached frame which is a non-inertia frame reference. In the body-attached frame, we assume the bead only subjects to the gravity force G_{bead}, the Stokes drag F_{d} and the inertial force F_{inertial}. We assume these forces are in equilibrium in every moment:

$$\begin{cases} F_{\text{d}} - G_{\text{bead}} - F_{\text{inertial}} = 0 \\ F_{\text{d}} = 6\pi\eta a\left(v_{\text{feeding}} - v_{\text{bead}}\right) \\ G_{\text{sphere}} = mg \\ F_{\text{inertial}} = ma_{\text{robot}} \\ m = \frac{4\pi a^3 \rho_{\text{bead}}}{3} \end{cases}, \tag{27}$$

where a, m, and ρ_{bead} are, respectively, the radius, mass, and density of the bead. η is the dynamic viscosity of the water. g is the standard gravity From Supplementary Equation 27, we can express v_{bead} as a function of v_{feeding}:

$$v_{\text{bead}} = v_{\text{feeding}} - \frac{2\rho_{\text{bead}}(g + a_{\text{robot}})a^2}{9\eta}. \tag{28}$$

According to Supplementary Equation 28, v_{bead} and v_{feeding} are linearly related. The larger the size of the bead is, the smaller the v_{bead} can be obtained with a given v_{feeding}. If $v_{\text{bead}}>0$, the bead catches up with the robot. Otherwise, the bead falls out through Mechanism-3. To illustrate this, the relationship between v_{bead} and v_{feeding} of the small and large beads are shown in Supplementary Figure 16a. We plot a horizontal axis along which $v_{\text{bead}}\equiv0$ (the black line). It intersects the blue line at point a and the red line at point b. The vertical black dashed lines passing through the point a and b separate the v_{feeding} axis into three regions.

In the region-i, neither the small nor large beads can catch up with the robot even when they are initially positioned inside the sub-umbrella region. There are two cases corresponding to the interval-i. In the first case, the v_{feeding} is too small to even levitate the beads upwards from the tank bottom. In the second case, the v_{feeding} is large enough to levitate beads upwards. However, since the beads are too slow to catch up with the robot, they eventually fall outside the sub-umbrella region through Mechanism-3. In the region-ii, the v_{feeding} further increases, enabling the small beads to catch up with the robot while leave the large beads behind. This is the mechanism applied in Mode D2. In Mode D2,

only the small beads can acquire high-enough speed to catch up with the robot and hence being transported. In the interval-iii, the v_{feeding} becomes strong enough to accelerate both the small and large beads to catch up with the robot, suggesting the cases in which both the small and large beads being trapped inside the bell.

When both the small and large beads can catch up with the robot, the Mechanism-3 can be neglected since the gravity of the beads is fully countered by the hydrodynamic force provided by the feeding flow. Therefore, the beads can escape the sub-umbrella region only through Mechanism-1 or 2. For the same swimming mode, small and large beads have different escaping probabilities (P_{C} and P_{R}) when circulating within the sub-umbrella region. This can be shown by their difference in relaxation time, which can be obtained by[17]:

$$\tau_{\mathrm{bead}} = \frac{2\rho_{\mathrm{bead}} a^2}{9\eta}, \tag{29}$$

where η is the dynamic viscosity of the water. The Supplementary Equation 29 implies the smaller beads have shorter relaxation time and hence follow the flow better than larger beads[17]. This means the smaller beads are more easily to be transported towards the lappet tip during the recovery phase, while the larger beads are more liable to stay close to the body central axis. After being transported away from the central axis, the beads can either be circulated out by Mechnism-2 or beaten out by Mechanism-1. Therefore, the small beads escape quickly while the large beads stay behind. This is the mechanism applied in Mode D1 to only transport the large beads while expelling the small beads.

Since the escaping through Mechanism-3 is closely related to the feeding flow velocity, understanding the relation between the kinematics and the feeding flow velocity is helpful in tuning this effect. In experiments, we use the average feeding flow velocity, $\overline{v}_{\mathrm{feeding}}$, as a metrics to quantify the strength of the feeding flow within one complete cycle of different swimming modes. $\overline{v}_{\mathrm{feeding}}$ is defined with the following equation:

$$\bar{v}_{\mathrm{feeding}} = \frac{\int_0^T (\bar{v}_{\mathrm{drift}}(t) - v_{\mathrm{robot}}(t))dt}{T}, \tag{30}$$

where $\overline{v}_{\mathrm{drift}}(t)$ is the drift flow velocity averaged along the reference line (the black dashed line in Supplementary Figure 9a). The $\overline{v}_{\mathrm{feeding}}$ of the five basic swimming modes are shown in Supplementary Figure 16b. In general, $\overline{v}_{\mathrm{feeding}}$ shows a similar trend as Q_{exchange} (Fig. 3c). To increase the velocity of the feeding flow, we can either decrease t_{C} to increase ω_{C} and induce higher v_{drift} (Mode B1) or decrease t_{R} to increase ω_{R} and enhance the downward motion of the robot during the recovery phase (Mode B2). To decrease the velocity of the feeding flow, we can decrease the θ_{R}, which is implemented to prescribe Mode-D2

Characterization and demonstration of Mode D1 and Mode D2. With the principles described above, we prescribe three kinematics to realize selective transportation. Mode D0 is used to collect beads under sub-umbrella region before the transportation. Mode D1 is used to only transport large beads while expelling the small beads. Mode D2 is used to only transport the small beads while leaving the large beads behind.

Before the transportation, the robot beats up and down with Mode D0 (Supplementary Figure 17) to collect beads inside the sub-umbrella region. To collect more beads, both the contraction and the recovery should be strong. However, t_C should not be shorter than t_R, or the robot would swim away from the tank bottom. Although both t_C and t_R should be as short as possible, we then choose t_C =75 ms and t_R= 50 ms (8 Hz) to make the beating frequency stay below to the cut-off frequency of the passive lappets (defined in section "Passive lappet" in "Method" and Supplementary Figure 2b). The 20 mT **B** field magnitude, which controls the beating amplitude (θ_R - θ_C), is experimentally chosen to suck in more beads while not significantly expelling them out, as the effect of beating amplitude on the object collection and object retaining is contradictory (Mode C in Table 1). The Mode D0 can be further optimized in the future.

Transporting large beads is realized by Mode D1. The robot first swims upwards from the bottom, then expells the small beads in the first few cycles while still keeping the large beads inside the sub-umbrella region. To let the robot swim upwards, we increase ω_C (58.05 rad·s^{-1}) and decrease ω_R (18.18 rad·s^{-1}) relative to Mode D0 by reducing t_C (26 ms) and increasing t_R (83 ms). Since ω_C has to be increased to a very high value so as to induce a strong enough feeding flow to make sure the large beads can always catch up the robot, here we increase ω_C by applying a step signal (Supplementary Figure 18a). A constant output after the contraction is applied to induce a glide phase for a longer retaining distance per cycle (Table 1, Fig. 4b). Mode D1 has different transportation capabilities to the small and large beads. Since the small beads are easily to be transported to the lappet tips during the recovery, P_C and P_R for small beads are higher than large beads, who are liable to stay near the body central axis during the recovery.

Transporting small beads is realized by Mode D2. Mode D2 is required to leave behind large beads and to enhance the retaining capability of the small beads. To achieve this, we decrease θ_R relative to Mode D1 (from 2.10 rad to 1.14 rad), by decreasing the maximum value of B_y (Supplementary Figure 18b). This effort can decrease the drift flow velocity while increasing the retaining capability according to Table 1.

The transportation performances of Mode D1 and Mode D2 to the small and large beads are quantified by experiments (Supplementary Figures 18c and 18d, Supplementary Movie 4). The experiments are conducted in a transparent water tank of size 100 × 60 × 40 mm^3 (length × width × height). In each trial, beads of the same size are piled on the tank bottom with the robot situated on the top. Before the transportation, the robot is controlled to beat twice by Mode D0 to collect beads into its sub-umbrella region. We then control the robot to swim with either Mode D1 or Mode D2 to transport only large or small beads. For comparison, we count the number of beads escaping at four height intervals that are separated with black dashed lines in Supplementary Figures 18c and 18d. With Mode D1, most of the small beads are expelled within the lowest height interval, while a large portion of the large beads can be transported to the uppermost height interval or even out of the field of view. With the Mode D2, most of the large beads are left within the lowest height

interval while a large portion of the small beads can be transported to the uppermost height interval or even out of the field of view.

We then mix the small and the large beads together to test whether the robot can still transport only the small or large target beads. We always place the large and small beads at the same initial position relative to the robot in each trial, with one large bead surrounded by small beads. The experiments demonstrate the selective transportation can be realized by using Mode D1 and Mode D2 (Fig. 7a, Supplementary Movie 4).

Investigating the influence of different factors on object manipulation performance. Finite element analysis is conducted using the commercial software COMSOL Multiphysics 5.3a (COMSOL, Inc., Stockholm, Sweden) to investigate the influence of the object size on object manipulation performance. The robot is modeled as a 2D beam (Supplementary Figure 19a), and its kinematics is prescribed based on the kinematics of Mode D1 and D2 extracted from experiments. The unsteady, incompressible, 2D Navier–Stokes equation is solved to obtain the velocity field of the fluid flow around the robot. The virtual bead used for tracing is assumed to have no effects on the surrounding flow field. The acceleration of the bead is assumed to be induced by the drag force acting on it. The drag force is modeled using Schiller-Naumann model. The flow velocity used to calculate the drag force is taken from the position where the circle center locates, therefore, the velocity distribution along the circumference is not considered. The computational area is set to be 100 × 40 mm^3 (width × height). The virtual robot starts to swim 3 mm above the 'tank' bottom (at 0 s in Supplementary Figure 19a). The virtual beads heavier than water (1050 $kg \cdot m^{-3}$) are initially set under the robot at the end of the first recovery phase (at 0.05 s in Supplementary Figure 19a). The computational time range is set to be 0.55 s, which covers three beating cycles of Mode D1 and D2. The collision force between the virtual beads is currently accounted as the linear elastic force.

We first set the diameters of the beads to be 0.5 mm and 1 mm to test whether the simulation model can capture the observations from the experiments. The results are shown in Supplementary Figure 19b. As expected, when the robot swims with Mode D1, the 1 mm beads follow after three beating cycles, while the 0.5 mm beads are quickly expelled within the second beating cycle. On the contrary, when the robot swims with Mode D2, the 1 mm beads are gradually left behind, while the 0.5 mm beads are still captured after three beating cycles. We then compare the transportation performances of the two kinematics regarding beads with four different diameters (0.25 mm, 0.5 mm, 1 mm, 2 mm) using the value of the normalized transporting height. The normalized transporting height is defined to be $H_{\mathrm{bead}}/H_{\mathrm{robot}}$, and the average of the two beads in each case is calculated. The closer the value is to 1, the better the beads can follow the robot, therefore, the better the transportation performance can be obtained. As shown in Supplementary Figure 19c, Mode D2 performs better on smaller beads (0.25 mm, 0.5 mm) while worse on larger beads (1 mm, 2 mm). Mode D1 performs better on 1 mm beads while worse on smaller beads (0.25 mm, 0.5 mm). However, if the bead becomes too large (*i.e.* 2 mm), Mode D1 cannot

produce strong enough flow to transport them upwards either. These results agree with the discussions on the transportation mechanism.

We also conduct the simulation to study whether the tank bottom has influences on object manipulation performance (Mode D1, D= 1mm). The initial position of the tank bottom is set to be 100 mm away from the initial position of the virtual robot. The initial position and the release time of the beads are all kept the same as described above. The results do not see a significant difference in transporting height of the beads although the flow field does show differences (Supplementary Figure 19d).

Moreover, we qualitatively demonstrate the strategy used for transporting spherical objects can also be applied to transport objects with various shapes through experiments (Supplementary Figure 20). Further quantitative investigations are needed to study the influence of the object shape on object manipulation performance.

Supplementary Note 13. Discussions on burrowing for camouflage and searching

The robot can hide into the fine beads or excavate target objects buried by mimicking the burrowing behaviors of many sea organisms[18,19]. For burrowing, the robot is required to stay at the tank bottom and transport beads from the below to the place above its body. To achieve this, we prescribe Mode E which has a very strong recovery. Specifically, we increases ω_R to 39.31 rad·s^{-1}, which is stronger when compared to all basic modes, by minimizing t_R ($\approx$ 10ms, which is equal to $5\times\tau$, where τ is the time constant of the coil system.) using a step signal, and maximize θ_R (2.63 rad) by increasing the maximum value of B_y to the upper limit of the coil system (30 mT). Additionally, the achieved θ_R cannot be further increased significantly even with a larger B_y since the magnetic core has reached its extreme deformation. A strong recovery can reduce the propulsion performance of the robot, increase the sub-umbrella circulation during the recovery and enhance Mechanism-2. The control signal used to realize Mode E can be found in Supplementary Figure 21a.

The burrowing process of the robot operating in Mode E is closely investigated by experiments (Supplementary Movie 5) which are conducted in a rectangular hole ($6 \times 6 \times 20$ mm^3 in length × width × depth) filled with fine polystyrene beads (200 ~ 300 μm in diameter, 1.05 g·cc^{-1} in density, Polysciences, Inc.). The robot is initially positioned on top of the beads. From the experiment, we find the beads can be transported away in two cases. In case-1 (Supplementary Figure 21b), the robot expels the trapped beads laterally during the recovery phase by Mechanism-2 and these beads do not fall back onto the robot body again. In case-2, the robot first expels the beads upwards by Mechanism-2 during the first recovery phase but these beads are not transported far away. Therefore, they fall back to the robot body during the contraction phase. During the second recovery phase, the robot can then beat these beads to a further place (Supplementary Figure 21c).

To realize camouflage, the robot is initially positioned flat. Therefore, it can expel the beads from the bottom to the place above its body. The expelled beads then gradually fall onto the robot and bury it. To realize object searching, however, the beads are expelled to a tilted direction to avoid burying the target objects again. Therefore, we position the robot with

an initial tilted angle and let the robot transport the beads out of the hole. With sustained burrowing, the target object, which is a stained polystyrene bead (diameter = 1 mm) that has been buried into the fine beads in advance, is finally dug out. The reader can refer to Supplementary Movie 5 for detailed information.

Supplementary Note 14. Discussions on enhancing local mixing

The robot can locally enhance the mixing of the fluid by using Mode F (Supplementary Figure 22a). To make the robot stay stationary and maximize the Mechanisms-1 and 2, we use symmetric signal here. Moreover, we prescribe Mode F with the maximum recovery angle ($\theta_R = 2.63$ rad) that can be achieved in our system by maximizing the peak and valley value of the magnetic field (30 mT). We also shorten both t_C and t_R to make the beating frequency 6 Hz. This frequency is below the cut-off frequency of the lappets based on our measurement (defined in section "Passive lappet" in "Method" and Supplementary Figure 2b) and can be further optimized in the future. Therefore, we obtain the enhanced angular velocities of the contraction ($\omega_C = 27.61$ rad·s^{-1}) and the recovery ($\omega_R = 27.70$ rad·s^{-1}). With such angular velocities, the robot stays at the tank bottom without swimming upwards. Under such frequency, the maximum θ_R achievable in our system (B_y capped by 30 mT) can help the robot to redistribute the mixed dye by enhancing Mechanism-2. Similarly, the enhanced ω_C can help the robot to redistribute the mixed dye by enhancing Mechanism-1 and to squeeze the dyes to the central axis to enhance the mixing under the bell.

The mixing process of Mode F is demonstrated in Supplementary Figures 22b and c and Supplementary Movie 6. The experiment is conducted in a water tank with a size 50 × 6 × 25 mm^3 (length × width × height). The food dyes with red and green colors are injected from both sides. The robot starts to mix when two dyes meet at the center bottom of the tank (at 0 s in Supplementary Figure 22b). The mixing is realized with the following steps. First, the robot draws in the red and green dyes from both sides during the recovery (at 0.08 s and 0.43 s in Supplementary Figure 22b). Secondly, the robot squeezes the dyes to the central axis during the contraction phase for mixing (at 0.19 s and 0.52 s in Supplementary Figure 22b). Lastly, the dyes mixed in the sub-umbrella region (indicated at 1.22 s in Supplementary Figure 22b) are expelled through Mechanism-1 and 2 (at 1.26 s and 15.77 s in Supplementary Figure 22b). The mixing mechanism is also summarized in Supplementary Figure 22c.

Supplementary Note 15. Discussions on creating the desired chemical path

The robot can create a chemical path in water with the wake structure induced during the propulsion. To qualitatively investigate the relationship between the propulsion kinematics and the wake structures, we visualize the wake structures created by five basic swimming modes using Fluorescein dye (Supplementary Figure 23b, Supplementary Movie 7). The experiment is conducted in a transparent water tank of size 100 × 60 × 40 mm^3 (length × width × height). The dye bolus is injected onto the robot's body when the robot rests on the tank bottom. When swimming with Mode A, a portion of the dye wrapping the robot's body gradually sheds, creating one layer of 8-fingered-skirt structure per cycle as depicted in real jellyfish ephyra[20]. When swimming with Mode B1, the dye tree becomes more

sparse. The dye shed from the robot body drifts downwards significantly due to the enhanced Mechanism-1. The stem connecting the adjacent two 8-fingered-skirt layers is elongated and narrowed down, which is due to the longer travel distance per cycle and higher propulsion velocity. When swimming with Mode B2, the dye structure becomes denser and more expanded relative to Mode A. The travel distance per cycle of Mode B2 is shortened, which makes the adjacent 8-fingered-skirt layers overlap each other. The Mechanism-2 is also enhanced in Mode B2, which makes more dye being expelled laterally during the recovery and hence horizontally inflates the dye tree structure. When swimming with Mode B3, the robot can produce dye tree structure that is similar to Mode A. However, due to the longer travel distance per cycle, the distance between two 8-fingered-skirt layers is increased. When swimming with Mode C, the dye tree structure becomes slender and concentrated. This is because Mode C has the best object retaining performance among five basic modes due to the small θ_R and can resist the spreading of the chemical. At last, it has to be noted that, compared to the swimming velocity achieved in pure water, the kinematics and velocity of the robot change slightly when swimming with the dye. This may due to the density and the viscosity change brought by the dye wrapping the robot body, and this phenomenon will be investigated in the future.

Since the propulsion velocity of Mode C is slow, we prescribe Mode G to increase the speed of creating the chemical path. The Mode G is prescribed by increasing the frequency and decreasing the valley value of the control signal of Mode C (Fig. S22a). Compared to Mode C, Mode G achieves a larger ω_C (7.53 rad·s^{-1}), a larger ω_R (6.66 rad·s^{-1}), a smaller θ_C (0.43 rad) and a smaller θ_R (0.78 rad). We decrease θ_C and θ_R here to create an even tighter sub-umbrella region to further restrain the spreading of the dye. We also add one more pair of coils horizontally in our setup to realize the steering in 2D for the purpose of creating a chemical path with a more complex shape. The experiment shows the robot can create a concentrated chemical path with an S shape (Fig. 7d, Supplementary Movie 7). Note that the optimization of the robot velocity and the chemical path is out of the scope of this current manuscript. This topic will be investigated in the future.

Supplementary References

1 Feitl, K. E., Millett, A. F., Colin, S. P., Dabiri, J. O. & Costello, J. H. Functional morphology and fluid interactions during early development of the scyphomedusa Aurelia aurita. *Biol. Bull.* **217**, 283-291 (2009).

2 Lynch, K. M. & Park, F. C. *Modern Robotics*. (Cambridge University Press, 2017).

3 Nawroth, J. C., Feitl, K. E., Colin, S. P., Costello, J. H. & Dabiri, J. O. Phenotypic plasticity in juvenile jellyfish medusae facilitates effective animal-fluid interaction. *Biol. Lett.* **6**, 389-393 (2010).

4 Nawroth, J. C., Lee, H., Feinberg, A. W., Ripplinger, C. M., McCain, M. L., Grosberg, A., Dabiri, J. O. & Parker, K. K. A tissue-engineered jellyfish with biomimetic propulsion. *Nat. Biotechnol.* **30**, 792-797 (2012).

5 Nagata, R. M., Morandini, A. C., Colin, S. P., Migotto, A. E. & Costello, J. H. Transitions in morphologies, fluid regimes, and feeding mechanisms during development of the medusa Lychnorhiza lucerna. *Mar. Ecol.-Prog. Ser.* **557**, 145-159 (2016).

6 Ceylan, H., Yasa, I. C., Yasa, O., Tabak, A. F., Giltinan, J. & Sitti, M. 3D-Printed Biodegradable Microswimmer for Theranostic Cargo Delivery and Release. *ACS Nano* **13**, 3353–3362 (2019).

7 Drucker, E. G. & Lauder, G. V. Locomotor forces on a swimming fish: three-dimensional vortex wake dynamics quantified using digital particle image velocimetry. *J. Exp. Biol.* **202**, 2393-2412 (1999).

8 McHenry, M. J. & Jed, J. The ontogenetic scaling of hydrodynamics and swimming performance in jellyfish (Aurelia aurita). *J. Exp. Biol.* **206**, 4125-4137 (2003).

9 Blough, T., Colin, S. P., Costello, J. H. & Marques, A. C. Ontogenetic changes in the bell morphology and kinematics and swimming behavior of rowing medusae: the special case of the limnomedusa Liriope tetraphylla. *Biol. Bull.* **220**, 6-14 (2011).

10 Masliyah, J. H. & Epstein, N. Steady Symmetric Flow Past Elliptical Cylinders. *Ind. Eng. Chem. Fund.* **10**, 293-299 (1971).

11 Roos, F. W. & Willmart.Ww. Some Experimental Results on Sphere and Disk Drag. *AIAA J.* **9**, 285-291 (1971).

12 Turton, R. & Levenspiel, O. A Short Note on the Drag Correlation for Spheres. *Powder Technol.* **47**, 83-86 (1986).

13 Lum, G. Z., Ye, Z., Dong, X., Marvi, H., Erin, O., Hu, W. & Sitti, M. Shape-programmable magnetic soft matter. *Proc. Natl. Acad. Sci.* **113**, E6007-E6015 (2016).

14 Zhao, R., Kim, Y., Chester, S. A., Sharma, P. & Zhao, X. Mechanics of Hard-Magnetic Soft Materials. *J. Mech. Phys. Solids* **124**, 244-263 (2018).

15 Boonvisut, P. & Çavuşoğlu, M. C. Estimation of soft tissue mechanical parameters from robotic manipulation data. *IEEE-ASME Trans. Mechatron.* **18**, 1602-1611 (2013).

16 Law, K. L. & Thompson, R. C. Oceans. Microplastics in the seas. *Science* **345**, 144-145 (2014).

17 Klinkenberg, J., de Lange, H. C. & Brandt, L. Modal and non-modal stability of particle-laden channel flow. *Phys. Fluids* **23**, 064110 (2011).

18 Hanlon, R. T., Watson, A. C. & Barbosa, A. A “mimic octopus” in the Atlantic: flatfish mimicry and camouflage by Macrotritopus defilippi. *Biol. Bull.* **218**, 15-24 (2010).

19 Able, K. W., Grimes, C. B., Cooper, R. A. & Uzmann, J. R. Burrow Construction and Behavior of Tilefish, Lopholatilus-Chamaeleonticeps, in Hudson Submarine-Canyon. *Environ. Biol. Fishes* **7**, 199-205 (1982).

20 Nawroth, J. C. & Dabiri, J. O. Induced drift by a self-propelled swimmer at intermediate Reynolds numbers. *Phys. Fluids* **26**, 091108 (2014).
