# Supplementary material for: Multi-functional soft-bodied jellyfish-like swimming
Source: Nat Commun. 2019 Jul 2;10:2703. doi: 10.1038/s41467-019-10549-7 (PMC6606650; doi:10.1038/s41467-019-10549-7)
Supplement: Supplementary file 3 — Description of Additional Supplementary Files [file 41467_2019_10549_MOESM3_ESM.pdf]

Description of Additional Supplementary Files

Supplementary Movie 1.
Biomimetic mode (Mode-A)

Supplementary Movie 2.
Kinematics and propulsion performance of five basic modes

Supplementary Movie 3.
Object retaining performance of five basic modes

Supplementary Movie 4.
Selectively transporting beads of two sizes

Supplementary Movie 5.
Burrowing for camouflage and object searching

Supplementary Movie 6.
Localized mixing

Supplementary Movie7.
Create dye path
